# MrpH, a new class of metal-binding adhesin, requires zinc to mediate biofilm formation

**DOI:** 10.1371/journal.ppat.1008707

**Published:** 2020-08-11

**Authors:** Wangshu Jiang, Wimal Ubhayasekera, Michael C. Breed, Allison N. Norsworthy, Nina Serr, Harry L. T. Mobley, Melanie M. Pearson, Stefan D. Knight

**Affiliations:** 1 Department of Cell and Molecular Biology, Uppsala University, Biomedical Center, Uppsala, Sweden; 2 Department of Microbiology and Immunology, University of Michigan Medical School, Ann Arbor, MI, United States of America; 3 Department of Microbiology, New York University School of Medicine, New York, NY, United States of America; University of Washington, UNITED STATES

## Abstract

*Proteus mirabilis*, a Gram-negative uropathogen, is a major causative agent in catheter-associated urinary tract infections (CAUTI). Mannose-resistant *Proteus*-like fimbriae (MR/P) are crucially important for *P*. *mirabilis* infectivity and are required for biofilm formation and auto-aggregation, as well as for bladder and kidney colonization. Here, the X-ray crystal structure of the MR/P tip adhesin, MrpH, is reported. The structure has a fold not previously described and contains a transition metal center with Zn^2+^ coordinated by three conserved histidine residues and a ligand. Using biofilm assays, chelation, metal complementation, and site-directed mutagenesis of the three histidines, we show that an intact metal binding site occupied by zinc is essential for MR/P fimbria-mediated biofilm formation, and furthermore, that *P*. *mirabilis* biofilm formation is reversible in a zinc-dependent manner. Zinc is also required for MR/P-dependent agglutination of erythrocytes, and mutation of the metal binding site renders *P*. *mirabilis* unfit in a mouse model of UTI. The studies presented here provide important clues as to the mechanism of MR/P-mediated biofilm formation and serve as a starting point for identifying the physiological MR/P fimbrial receptor.

## Author summary

Many bacteria use fimbriae to adhere to surfaces, and this function is often essential for pathogens to gain a foothold in the host. In this study, we examine the major virulence-associated fimbrial protein, MrpH, of the bacterial urinary tract pathogen *Proteus mirabilis*. This species is particularly known for causing catheter-associated urinary tract infections, in which it forms damaging urinary stones and crystalline biofilms that can block the flow of urine through indwelling catheters. MrpH resides at the tip of mannose-resistant *Proteus-*like (MR/P) fimbriae and is required for MR/P-dependent adherence to surfaces. Although MR/P belongs to a well-known class of adhesive fimbriae encoded by the chaperone-usher pathway, we found that MrpH has a dramatically different structure compared with other tip-located adhesins in this family. Unexpectedly, MrpH was found to bind a zinc cation, which we show is essential for MR/P-mediated biofilm formation and adherence to red blood cells. Furthermore, MR/P-mediated adherence can be modified by controlling zinc levels. These findings have the potential to aid development of better anti-biofilm urinary catheters or other methods to prevent *P*. *mirabilis* infection of the urinary tract.

## Introduction

The urinary tract is a primary target for bacterial infections [1]. Clinically, urinary tract infections (UTIs) are categorized as uncomplicated or complicated. Uncomplicated UTIs occur in individuals that are otherwise healthy, with uropathogenic *Escherichia coli* (UPEC) as the main causative agent [2,3]. Complicated UTIs affect patients with underlying difficulties such as indwelling catheters or anatomic obstructions. In particular, catheter-associated UTIs (CAUTI) are one of the most common healthcare-associated infections [4,5]. *Proteus mirabilis*, a Gram-negative member of the *Enterobacterales* bacterial order famous for its ability to swarm over surfaces, including urinary catheters, is a major causative agent in CAUTI [6–8].

*P*. *mirabilis* utilizes a multitude of virulence factors including urease, flagella, toxins, and fimbriae, to establish and promote infection [9,10]. Even in the absence of catheterization, *P*. *mirabilis* UTI is self-complicating, because potent urease activity hydrolyzes urea, leading to an increase in urinary pH and subsequent stone formation (urolithiasis) [11,12]. In the presence of a urinary catheter, the combined actions of bacterial adherence and urease activity lead to the formation of crystalline biofilms consisting of surface-associated bacterial communities embedded in struvite and apatite crystals [13–15]. Another notable feature of *P*. *mirabilis* is its diverse collection of fimbrial genes, with 17 distinct operons encoded by the type strain HI4320 [16,17]. The receptors for most of these fimbriae remain unknown.

**Fig 1:**
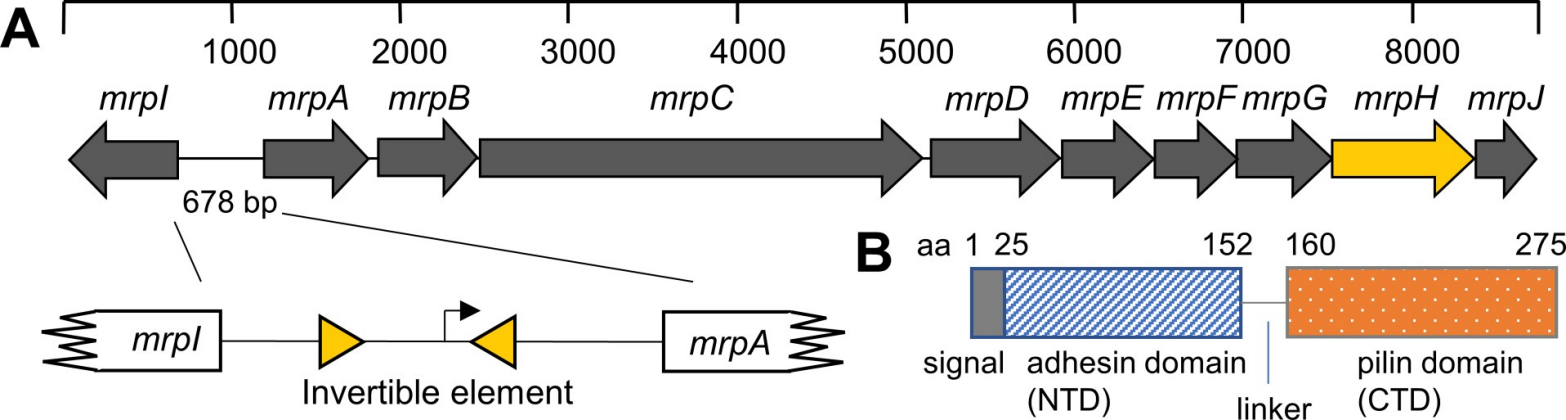
MR/P operon and MrpH domain organization. (A) The genes encoding MR/P fimbriae are organized as an operon. An invertible element (IE) in the *mrp* promoter controls *mrp* expression (inset), and MrpI is the recombinase that flips the IE. Therefore, *mrpI* mutants are either locked “ON” or “OFF” for MR/P fimbriae. The tip-located TDA is encoded by *mrpH* (yellow). (B) Depiction of linear MrpH. The predicted N-terminal domain (NTD) was used for protein crystallization. Because the C-terminal domain (CTD) has not yet been crystallized, the beginning residue shown here is an estimate.

Mannose-resistant *Proteus*-like fimbriae (MR/P) are the most extensively studied *P*. *mirabilis* fimbriae. MR/P fimbriae are required for biofilm formation and autoaggregation, and expression is essential for bladder and kidney colonization [18,19]. MR/P fimbriae are expressed from the *mrpABCDEFGHJ* operon (Fig 1A), where *mrpA* encodes the major structural subunit, *mrpC* the outer membrane usher, *mrpD* the periplasmic chaperone, *mrpB* and *mrpE-G* four minor subunits, *mrpH* the tip-located adhesin, and *mrpJ* a transcriptional regulator [20]. MrpH is a two-domain adhesin (TDA) consisting of an N-terminal receptor binding domain (NTD) joined through a short linker to a C-terminal pilin domain that attaches the TDA to the fimbrial tip (Fig 1B) [21].

Compared with *in vitro* culture, *mrp* genes are the most highly induced of all genes in *P*. *mirabilis* bacteria collected from urine of infected mice [22]. The *mrp* operon is sufficient for MR/P production and assembly, and expression of a vector containing the *P*. *mirabilis* HI4320 *mrpABCDEFGH* genes in *Escherichia coli* DH5α leads to the formation of MR/P fimbriae and hemagglutination activity [23]. Isogenic *P*. *mirabilis mrp* mutants show significant deficiency in colonization of bladder and kidney, particularly after longer-term (1 week) or in cochallenge competition experiments [23–27]. Compared to wild-type *P*. *mirabilis*, an *mrpA* mutant failed to develop the extracellular bacterial clusters essential for bladder stone formation, a hallmark of *P*. *mirabilis* UTI [19]. Neither a *P*. *mirabilis* HI4320 *mrpH* knockout, nor *E*. *coli* DH5α encoding all of the *mrp* genes except *mrpH*, is able to produce normal MR/P fimbriae or hemagglutinate red blood cells [23]. Loss of hemagglutination activity in the absence of MrpH can be explained by the inability to properly assemble MR/P fimbriae in the absence of the TDA. However, mutating two cysteine residues involved in disulfide bond formation in MrpH to serine also abolishes the ability to hemagglutinate even though MR/P fimbriae are produced. This provides direct evidence that MrpH is the adhesin responsible for binding to erythrocytes [23].

A second *mrp* transcript independently encodes a recombinase (MrpI) that dictates the expression of MR/P fimbriae via an invertible element (IE) containing the σ^70^ *mrp* operon promoter [20,28] (Fig 1A). Mutations in the *mrpI* gene result in *P*. *mirabilis* mutants constitutively expressing MR/P fimbriae (locked-ON or ‘L-ON’) or devoid of MR/P fimbriae (locked-OFF or ‘L-OFF’) [20]. Phase-locked constructs are particularly useful for studying MR/P function *in vitro*, when *mrp* genes are typically not well expressed (exceptions include culture in a hypoxic chamber maintained at 5% O_2_ [29] or serial passages of 3 x 48 h static cultures [24]). In a study by Jansen and colleagues [30], *P*. *mirabilis* L-ON formed significantly more biofilm during culture in urine than either the wild type or a *P*. *mirabilis* L-OFF mutant, which both formed similar levels of biofilms. However, wild-type biofilms were much thicker than those produced by L-ON or L-OFF bacteria. During short-term independent challenge experiments (1–4 days post-inoculation), although L-OFF *P*. *mirabilis* colonized mouse bladders to the same extent as wild type and L-ON bacteria, they preferentially located to areas of exfoliation instead of intact uroepithelial umbrella cells, showing that MR/P fimbriae are important for determining tissue tropism [30]. A second kind of *P*. *mirabilis* fimbriae, ambient temperature fimbriae (ATF) [31,32], was identified on the surface of L-OFF bacteria attached to lamina propria [30]. Likely, MrpH is responsible for binding to an as of yet unidentified surface receptor on uroepithelial umbrella cells, while the ATF TDA AtfE might recognize sulfated glycosaminoglycans exposed on exfoliated bladder sections [33].

Although from a multitude of studies it is clear that MR/P fimbriae contribute significantly to UTI, the precise roles of MR/P fimbriae and their contributions to pathogenesis will remain elusive until their binding target is identified. Here, we report the crystal structure of the NTD of MrpH at 1.02 Å resolution, which reveals a divalent metal (Me^2+^)-binding site composed of three conserved histidine residues that might form (part of) a receptor binding site. We further show that an intact Me^2+^-binding site occupied by Zn^2+^ is required for MR/P-mediated biofilm formation and hemagglutination, and that biofilm *vs*. planktonic growth of *P*. *mirabilis* can be regulated by modulation of Zn^2+^ levels.

## Results

### The adhesin domain of MrpH has a distinctive fold

**Fig 2:**
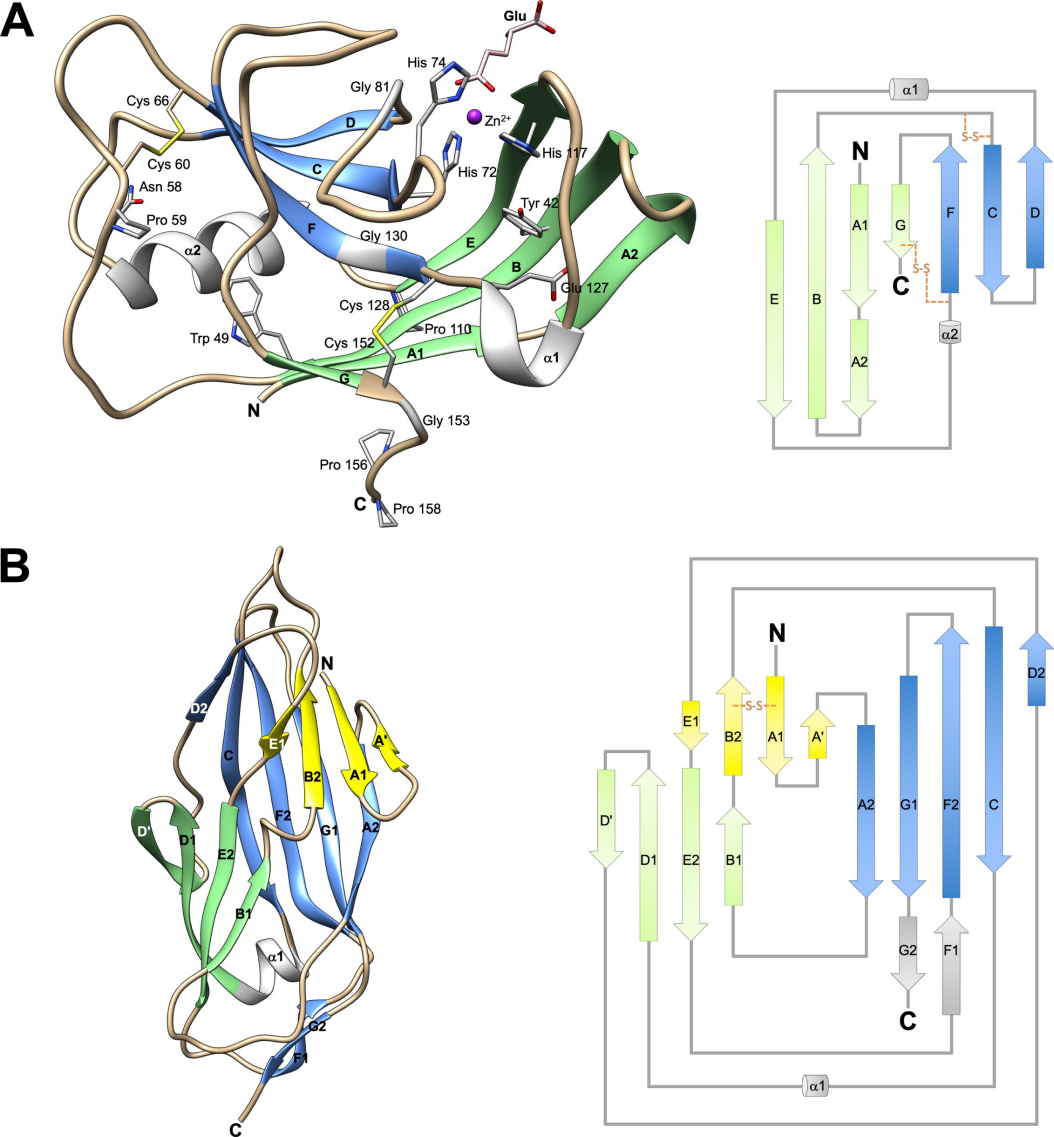
Three-dimensional crystal structure of MrpH_ntd_. (A) Cartoon representation (left) and topology diagram (right) of MrpH_159_. β-strands are labelled A-G from the N-terminus to the C-terminus. β-strands forming a β-sheet are in the same color: β-strands E, B, A and G are in green, β-strands F, C and D are in blue. Helices α1 and α2 are colored grey. Coils are colored tan. Highly conserved residues (carbon atoms in grey) identified by multiple sequence alignment (Fig 3), a non-conserved disulfide (carbons in tan), and the bound glutamic acid molecule (carbons in pink) are shown as sticks. Zn^2+^ is shown as a purple sphere. (B) Cartoon representation (left) and topology diagram (right) of the canonical fimbrial NTD of FimH (pdb id 1uwf) using the same coloring scheme as in (A).

**Fig 3:**
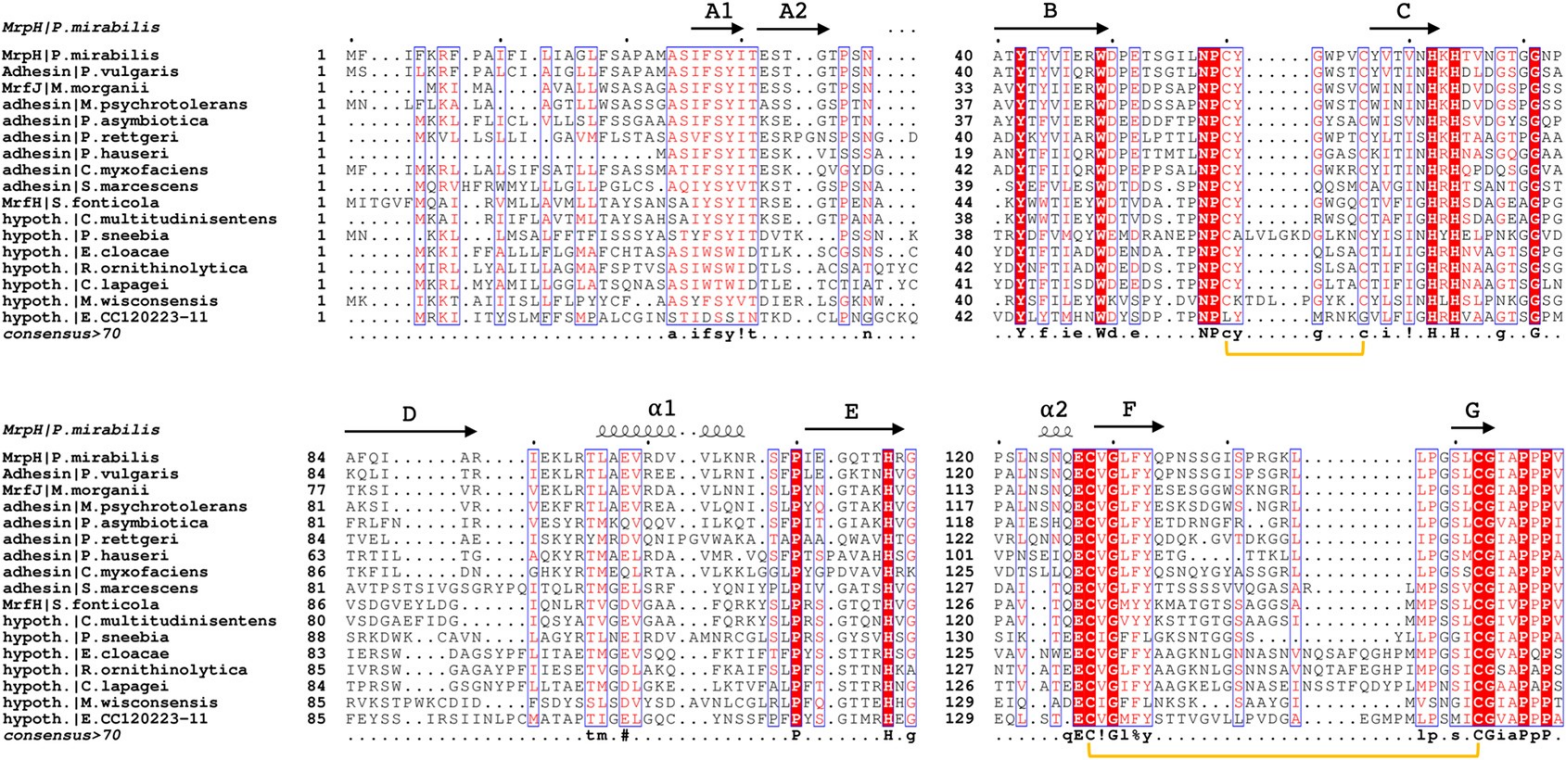
Multiple sequence alignment of representative MrpH_ntd_ amino acid sequences. Aligned MrpH_ntd_ amino acid sequences from 16 different organisms, with residues numbered according to their position in the native sequence. Identical residues are highlighted with red shading and white text, and similar residues with a blue frame and red text. The top secondary-structure depiction and bottom disulfide bond indications (shown as yellow brackets) are derived from the MrpH_ntd_ structure.

An established approach for overcoming the intrinsic instability of pilin domains to obtain material for structural and functional studies of TDAs is to express the receptor-binding NTDs by themselves [33–35]. Since the domain border cannot be precisely predicted, several constructs encoding the predicted NTD of MrpH (MrpH_ntd_) were generated (S1 Table). Two constructs yielding soluble protein, designated MrpH_153_ and MrpH_159,_ were used for crystallography. The structure of MrpH_153_ was solved to 1.02 Å resolution using single anomalous dispersion, and the structure of the longer MrpH_159_ construct solved to 1.75 Å by molecular replacement using MrpH_153_ as the search model. Data collection and refinement statistics are summarized in Table 1. The two structures are very similar, with an r.m.s.d. of 0.45 Å between 129 Cα atoms (Fig 2A, S1 Fig). In both structures, the first residue visible in the electron density maps corresponds to the first residue after the predicted signal sequence (Ser 25). The MrpH_ntd_ fold is distantly related to that of canonical TDAs such as FimH, UcaD, or AtfE [33,34], but has a distinct fold not previously observed (Fig 2A). The seven β-strands in MrpH_ntd_ (denoted A-G) are significantly shorter than in other TDAs, giving the structure a more compact shape with approximate dimensions of 45 Å x 25 Å x 27 Å. Compared to canonical TDAs (Fig 2B), the D1 strand is missing, and strands A and G have switched sheets to form one E-B-A-G and one F-C-D sheet. Also in contrast to other TDAs, the N- and C-termini are located relatively close to each other on one side of the molecule instead of on opposite sides. A Dali search [36] for similar structures produced no hits. A BLAST [37] search revealed the presence of MrpH-like proteins in a broad range of *Enterobacterales* (Fig 3).

**Table 1:** Native data collection and refinement statistics.

	MrpH_153_	MprH_159_
Data collection		
Space group	P2_1_	P2_1_2_1_2_1_
*a*, *b*, *c* (Å)	25.56, 53.14, 40.16	26.66, 54.49, 79.96
*α*, *β*, *γ* (°)	90.0, 102.84, 90	90, 90, 90
Molecules in a.u.	1	1
Wavelength (Å)	0.979	0.972
Resolution (Å)	31.52–1.02 (1.056–1.02)	18.85–1.75 (1.813–1.75)
Total reflections	241633 (20712)	78202 (7707)
Unique reflections	52285 (5020)	12317 (1215)
Multiplicity	4.6 (4.1)	6.3 (6.4)
Completeness (%)	98.22 (95.05)	98.97 (93.75)
*<I/σ(I)>*	15.00 (2.31)	12.70 (2.12)
Wilson B-factor (Å^2^)	9.17	11.18
*R*_*merge*_ ‡	0.0461 (0.549)	0.108 (0.563)
*R*_*meas*_ †	0.0516 (0.629)	0.118 (0.614)
*R*_*pim*_ §	0.0227 (0.300)	0.046 (0.242)
*CC*_*1/2*_	0.999 (0.878)	0.997 (0.934)
Refinement		
Number of reflections in work set	49705 (4800)	11593 (1091)
Number of reflections in test set	2563 (213)	631 (49)
*R*_*work*_ #	0.1077 (0.2251)	0.1528 (0.3013)
*R*_*free*_ #	0.1292 (0.2596)	0.1825 (0.2719)
Number of non-hydrogen atoms	1293	1257
protein	1121	1063
solvent	161	183
Ligands	1 Zn^2+^, 1 tartrate	1 Zn^2+^, 1 glutamate
R.m.s. deviations		
bonds (Å)	0.010	0.003
angles (°)	1.07	0.70
Ramachandran favored (%)	98.47	98.48
Ramachandran allowed (%)	1.53	1.52
Ramachandran outliers (%)	0.00	0.00
Rotamer outliers (%)	2.27	0.00
Clashscore	0.89	0.00
Average B-factor (Å^2^)	13.54	13.75
protein	12.04	12.37
ligands	16.44	23.35
solvent	23.76	21.83
Number of TLS groups	0	4
PDB code	6Y4E	6Y4F

‡ *R*_*merge*_ = $\sum_{hkl}\sum_{i}\left|I_{i}(hkl)-\langle I(hkl)\rangle \right|/\sum_{hkl}\sum_{i}I_{i}(hkl)$

† *R*_*meas*_ = $\sum_{hkl}\sqrt{\frac{n}{n-1}}\sum_{i}^{n}\left|I_{i}(hkl)-\langle I(hkl)\rangle \right|/\sum_{hkl}\sum_{i}I_{i}(hkl)$

§ *R*_*p*.*i*.*m*._ = $\sum_{hkl}\sqrt{\frac{1}{n-1}}\sum_{i}^{n}\left|I_{i}(hkl)-\langle I(hkl)\rangle \right|/\sum_{hkl}\sum_{i}I_{i}(hkl)$

*# R* = $\sum_{hkl}\left|F_{hkl}^{obs}-F_{hkl}^{calc}\right|/\sum_{hkl}F_{hkl}^{obs}.$ *R*_*work*_ is calculated for the work set of reflections used in refinement, *R*_*free*_ is calculated for a test set comprising 5% of the reflections.

The final G strand, instead of running along the length of the domain as in other TDAs, ends near the middle of the domain, at residue Cys 152 (Fig 2A), which forms a conserved disulfide bond with Cys 128. The C-terminal segment, essentially corresponding to the His-tag in MrpH_153_, and to the six last MrpH residues in MrpH_159_, turns almost 90° away from the path of the G strand, suggesting a hammerhead orientation of the domain with respect to the fimbrial axis, in contrast to the in-line orientation of other adhesin NTDs. This feature is probably conserved in all MrpH-like proteins since the linker region following Cys 152 consists of a conserved 153-GIAPPP-158 motif (Fig 3).

The disulfide bond is important for MR/P function, since mutation of either Cys 128 or Cys 152 to serine abolished hemagglutination [23]. Cys 152 defines the end of the globular domain, and the Cys 128—Cys 152 disulfide might be important for maintaining structural integrity, in particular, under shear force. In addition to the strictly conserved Cys 128—Cys 152 S-S bond, a second highly conserved disulfide bond, between Cys 60 and Cys 66, is present in the structure.

### MrpH is a zinc-binding protein

**Fig 4:**
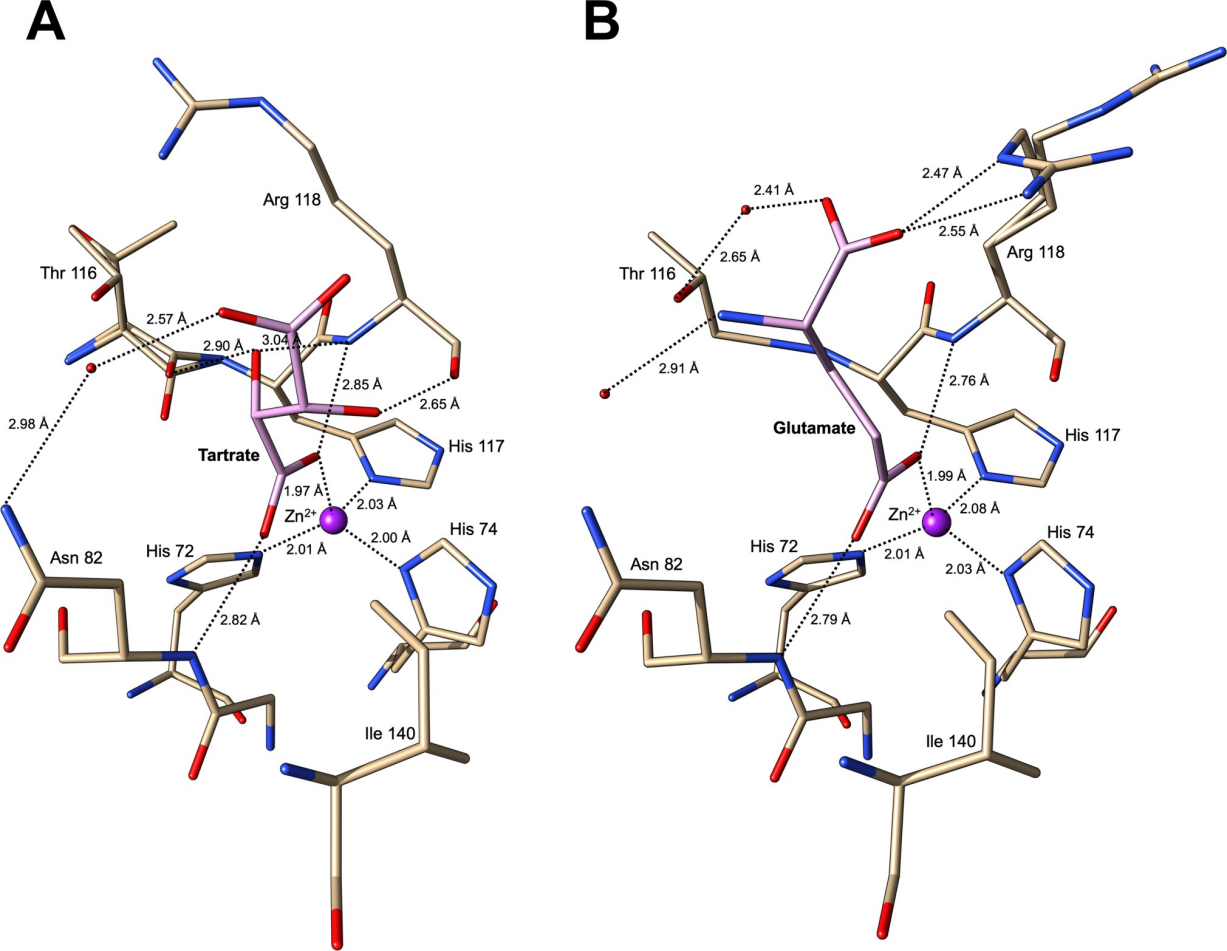
Close-up of the metal and ligand binding site in MrpH_ntd_. (A) MrpH_153_ ligand binding site with tartrate bound. (B) MrpH_159_ ligand binding site with glutamate bound. In both (A) and (B), residues interacting with the ligand or with the zinc ion are shown as sticks and labelled. Zinc is shown as a purple sphere. Water molecules are shown as red spheres. Hydrogen bonds and bonds to the zinc are shown as dotted lines with bond distances indicated next to the lines.

In both MrpH_153_ and MrpH_159_, a metal bound by three histidine (His 72, His 74, His 117) side chains was found (Fig 4, S2 Fig). The metal is tetrahedrally coordinated by the Nε nitrogen of His 72, the Nδ nitrogens of His 74 and His 117, and by an external ligand (see below). The binding site resembles a classical zinc binding site as found in α-, γ- and δ-carbonic anhydrases [38]. Crystallographic refinement trials with different transition metals (Mn, Fe, Co, Ni, Cu, Zn) clearly indicated Cu or Zn as the bound metal, both in terms of the crystallographic residual (*R*_*free*_) and m*Fo*-D*Fc* difference electron density. Running the structures through the *CheckMyMetal* server [39] validated the choice of Zn or Cu as the bound metal. Neither zinc nor copper was added during protein expression, purification or crystallization, suggesting that the metal was picked up during expression in *E*. *coli*. We used electroparamagnetic resonance (EPR) and total reflection x-ray fluorescence (TXRF) spectroscopy to identify the metal bound to MrpH. While no EPR signal was detected using native protein sample, excluding copper as the bound metal (EPR gives a strong signal for copper whereas zinc is silent), the TXRF spectrum (S3 Fig) showed a strong zinc signal, confirming MrpH as a zinc-binding protein. Based on this and on follow-up experiments presented below, we have modelled a Zn^2+^ ion in the metal binding site. Transition metal binding is likely a conserved feature of all MrpH-like adhesins, since the three metal-coordinating histidines are strictly conserved (Fig 3).

### Identification of a possible receptor-binding site

While optimizing crystallization conditions for MrpH_153_, we carried out thermal shift assay (TSA) experiments [40] to identify conditions that would stabilize the protein and promote crystallization. A melting temperature (T_m_) shift from 66.7 ± 0.6°C in 10 mM HEPES buffer, pH 7.5, to 78.5 ± 0.1°C in 10 mM ammonium tartrate pH 7.0, 100 mM NaCl, was observed. A similar result was obtained in TSA experiments using the longer MrpH_159_ construct, where 10 mM tartrate increased the T_m_ from 65.2 ± 0.3°C to 80.1 ± 0.7°C (Table 2).

**Table 2:** Results of TSA experiments.

Condition	<T_m_> (°C)^*a*^
Storage buffer	65.2±0.30
Ammonium tartrate	80.1±0.70
Sodium citrate	73.3±0.64
Sodium acetate	76.3±0.56
Sodium formate	68.4±0.75
Sodium salicylate	65.2±0.02
Sodium oxamate	66.6±0.62
Glutamic acid	73.1±0.21
Glutamine	66.1±0.17
Aspartic acid	65.5±0.11
Asparagine	65.6±0.18
Sodium citrate pH 5.5	73.0±0.06
Sodium cacodylate pH 6.5	72.7±0.78
MES pH 6.5	69.1±0.31
Potassium phosphate pH 6.5	71.7±0.06
PIPES pH 7.0	72.0±0.05
Imidazole pH 7.0	66.2±0.10
MOPS pH 7.0	67.1±0.27
Potassium phosphate pH 7.5	69.7±0.18
DIPSO pH 7.5	68.4±0.10
Tricine pH 7.5	66.3±0.07
HEPES pH 8.0	65.3±1.11
Tris pH 8.0	65.5±0.05
Tris pH 8.5	65.2±0.06
CHES pH 9.0	65.4±0.38
Glycine pH 9.5	65.4±0.15

^*a*^ Average melting temperature (<T_m_>) ± one standard deviation from three replicates.

Based on the TSA results, ammonium tartrate was added to the MrpH_153_ protein solution prior to crystallization. The m*Fo*-D*Fc* difference electron density maps after completion of the protein models and placing waters suggested that a ligand was bound to the Zn^2+^, both in MrpH_153_ and in MrpH_159_ (S2 Fig). Consistent with the significant stabilization of MrpH_153_ by tartrate in the TSA experiments, a molecule of 1(+)-tartaric acid could be nicely fitted into the electron density for MrpH_153_ (S2A Fig), leading to an improved model as indicated by a drop in *R*_*free*_ of 0.8%-units. One of the tartrate carboxyl oxygens (O1) ligates the Zn^2+^, completing the tetrahedral co-ordination sphere around the metal, and in addition forms a hydrogen bond to the main chain nitrogen of Arg 118 (Fig 4A). The second oxygen (O11) from the same carboxyl group forms a hydrogen bond to the main chain nitrogen of Asn 82. Both hydroxyl groups of the tartrate molecule are also involved in hydrogen bond interactions with MrpH_153_ main chain atoms: O2 with Arg 118 N and the carbonyl oxygen of Thr 116, and O3 with the carbonyl oxygen of Arg 118. These interactions would appear to provide at least some specificity since other carboxylic acids such as formate, acetate, or citrate did not provide the same level of stabilization as tartrate, as measured by TSA (Table 2).

Similar electron density, consistent with the binding of a carboxyl group ligand, was present also in the MrpH_159_ difference electron density map (S2B Fig). No tartrate was included in MrpH_159_ storage buffer or crystallization conditions, and tartrate did not fit the density. We therefore concluded that an unknown ligand, possibly a (di)carboxylic acid, had been picked up by MrpH_159_ during protein expression. Since our TSA experiments showed stabilization of MrpH_159_ by glutamic acid (increasing the T_m_ by 7.9°C), but not by glutamine, aspartic acid, or asparagine (Table 2), we decided to test if glutamic acid could explain the difference electron density. We found that a molecule of glutamate could convincingly be fitted in the density with the side chain carboxyl group ligating the zinc ion (S2B Fig), resulting in a 1.4%-unit R_free_ decrease following refinement, so we have assigned glutamic acid as the bound ligand in the MrpH_159_ structure. In addition to the carboxyl group interactions with the protein, which are the same as for tartrate, there is also a water-mediated hydrogen bond interaction with the side chain of Asn 82, and a salt bridge from the Glu main chain carboxylate to the guanidinium group of Arg 118 (Fig 4B).

**Fig 5:**
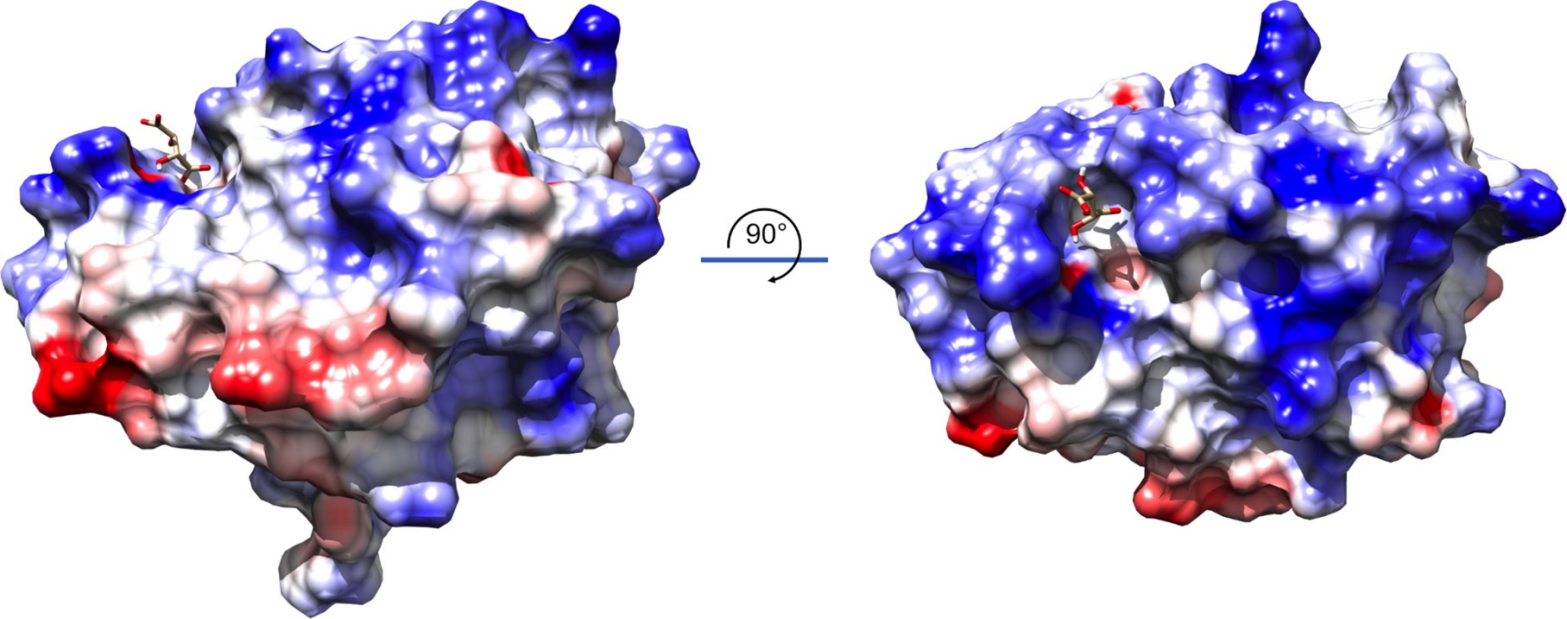
The top surface of MrpH_ntd_ is highly positively charged. Side (left) and top (right) views showing the electrostatic surface of MrpH_ntd_. A crevice extending from the ligand-binding site (tartrate displayed as a stick model) could serve as a binding site for an extended, possibly negatively charged, ligand and/or receptor. Alternatively, the positively charged surface could facilitate approach to negatively charged surfaces without being directly involved in binding. Positive surfaces are blue, neutral surfaces are white, and negative surfaces are red.

The “top” surface of MrpH_ntd_, where the ligand binding site is located, is positively charged (Fig 5). A small crevice extends from the ligand binding site and down toward the equator of the molecule. These intriguing features might provide surfaces for binding of a more extended, possibly negatively charged, receptor.

### Biofilm formation is MrpH-dependent and enhanced at acidic pH

**Fig 6:**
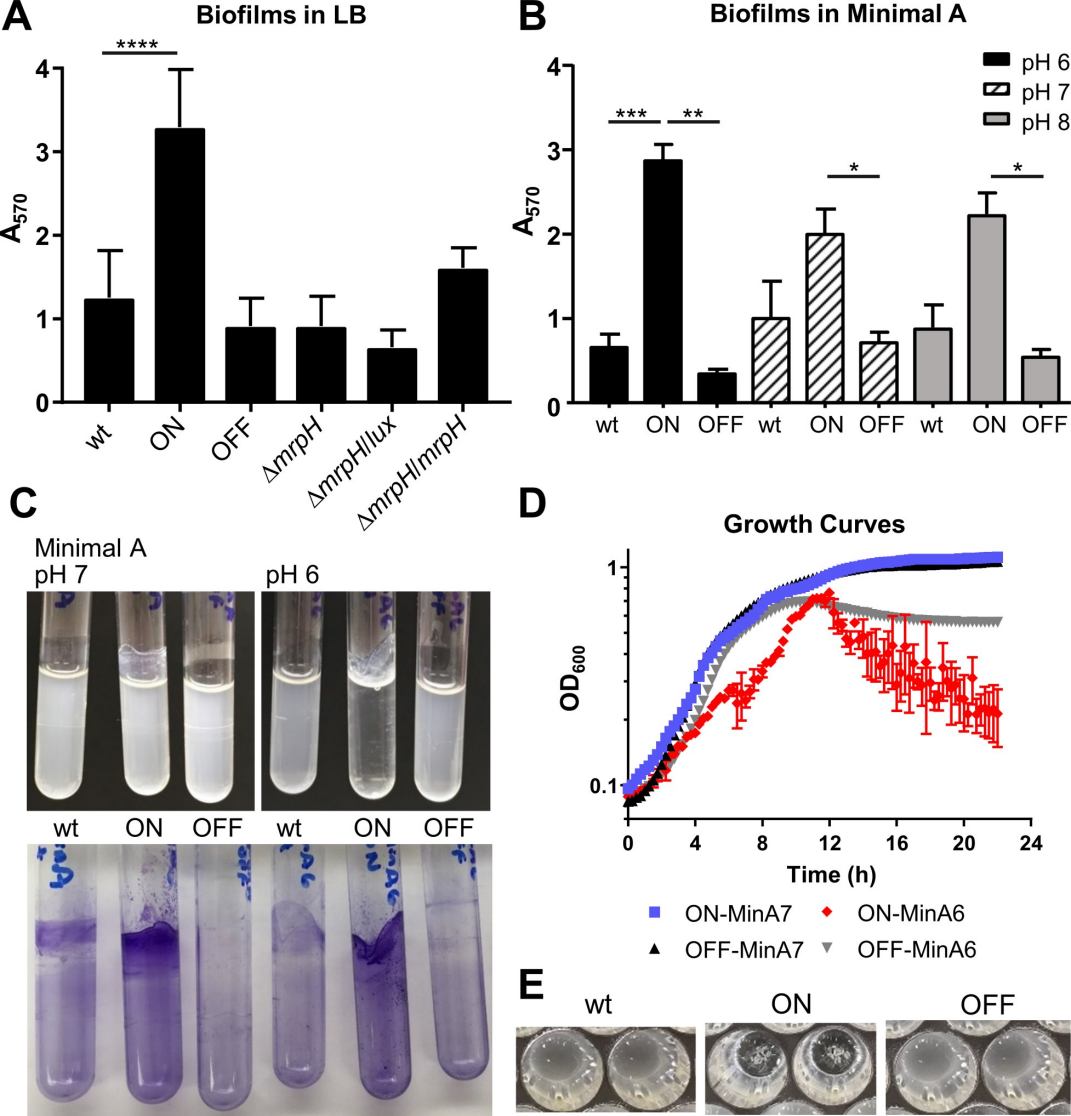
Biofilm formation is dependent on MR/P fimbriae and pH. (A) Biofilm formation in LB medium. MR/P locked-ON formed significantly more biofilm than all other tested strains. Wild-type biofilm formation trended higher than MR/P locked-OFF, but the difference was not significant. The last three columns show an *mrpH* mutant (Δ*mrpH*), either without a plasmid or complemented with pGEN-Pmrp-*lux* or pGEN-Pmrp-*mrpH*. (B-D) Biofilm formation was more pronounced at acidic pH. (B) Quantification of biofilm formation in Minimal A. Significance calculated using one-way ANOVA with Dunnett’s multiple comparisons test. (C) Representative biofilm cultures (top) and biofilm staining (bottom). (D) Growth curve analysis of *P*. *mirabilis* HI4320 L-ON and L-OFF at pH 6 and 7. (E) Growth curve wells showing floating pellicles for L-ON and planktonic growth for wild type and L-OFF.

The structure of recombinant MrpH_ntd_ suggested a possible role for zinc in *P*. *mirabilis* MR/P fimbrial function. MR/P-dependent biofilm formation has been previously reported [30,41,42]. Therefore, we designed experiments to test the role of divalent metal ions and the significance of the MrpH Zn^2+^-binding site for MR/P-mediated biofilm formation in a native *P*. *mirabilis* background. Culture of *P*. *mirabilis* in a relatively rich complex medium, LB, resulted in modest biofilm formation by wild-type HI4320 that was not statistically significantly better than an isogenic *mrpI* mutant that was MR/P locked-OFF (Fig 6A; wt vs OFF). During UTI, *P*. *mirabilis* urease activity results in alkaline urinary pH, and we reasoned that this might be conducive to biofilm formation. Instead, when biofilm assays were conducted in a minimal, chemically-defined medium (Minimal A [43]), we found enhanced biofilm formation by MR/P locked-ON bacteria at pH 6 with very little background from the isogenic locked-OFF mutant (Fig 6B). The biofilm switch was so dramatic that the vast majority of the bacteria were not in the planktonic phase (Fig 6C), which resulted in distinctive growth curve measurements due to the formation of floating mats of bacteria (*i*.*e*., pellicles; Fig 6D and 6E) on top of very low turbidity in the culture medium. Subsequent biofilm assays were conducted using this medium.

To investigate if pH affects MrpH_ntd_ stability, we tested the effect of different buffers at different pH values on the melting temperature of MrpH_159_. Interestingly, we observed a linear increase in T_m_ from about 65°C at pH 8 and above to 73°C at pH 5.5 (Table 2, S4 Fig), indicating that the protein was more stable at acidic pH values.

**Fig 7:**
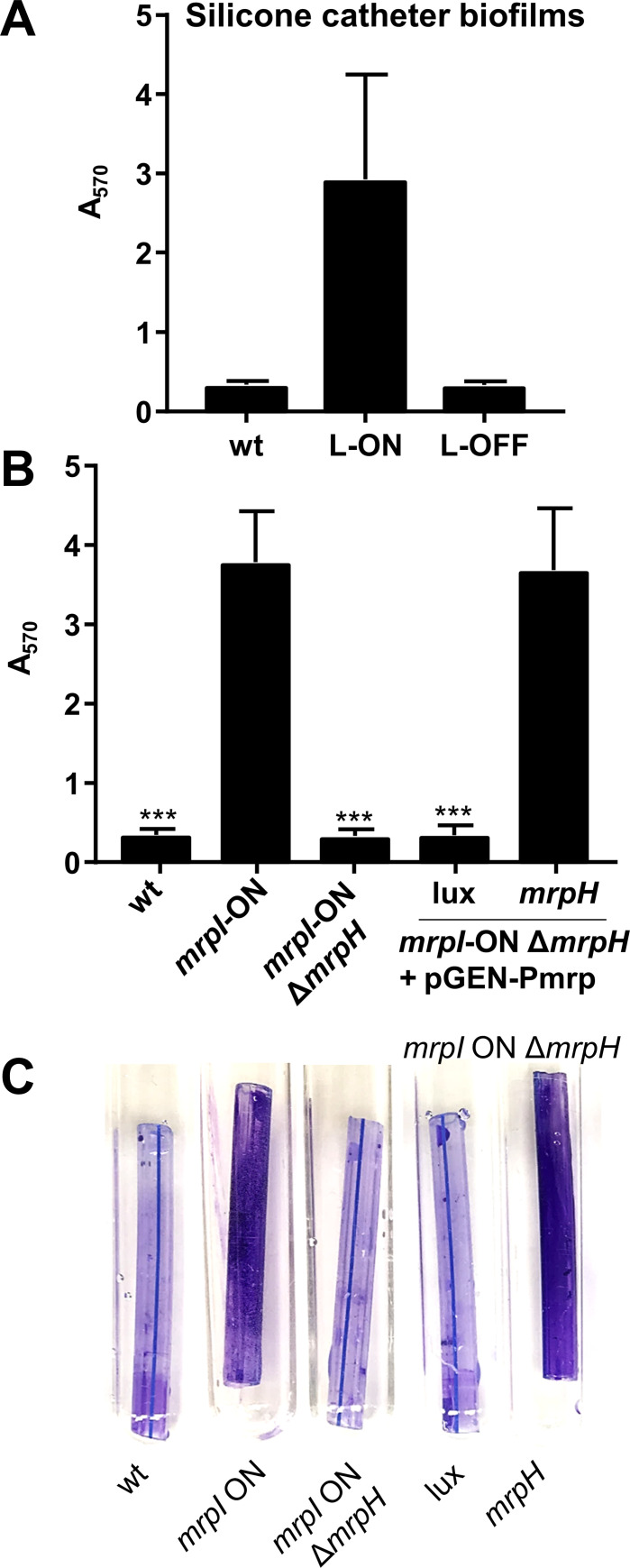
MrpH-dependent biofilm formation occurs on urinary catheter segments. Pieces of a silicone Foley catheter were added to culture tubes containing Minimal A, pH 6. Medium was inoculated 1:100 with overnight culture of *P*. *mirabilis*, and cultures were aerated at 37C for 24 h. Catheter segments were stained with crystal violet, then removed to a new tube for extraction and quantification of stain. (A) biofilm formation by *P*. *mirabilis* HI4320 (wt), L-ON, and L-OFF strains shown in previous figures (n = 3). (B) biofilm formation by an independently constructed locked ON strain (*mrpI*-ON), a double mutant (*mrpI*-ON Δ*mrpH*), and the double mutant complemented with a plasmid containing either luciferase (lux) or *mrpH*, both under the native *mrp* operon promoter (P*mrp*) (n = 5–6). Statistical significance was assessed *versus mrpI*-ON. (C) a representative example of biofilm formation by the strains shown in B.

Furthermore, we found MR/P-dependent biofilm formation on segments of silicone urinary catheters (Fig 7). This is particularly relevant because *mrp* genes are strongly induced during experimental UTI in mice, with a population of bacteria that is nearly all *mrp* ON (that is, with the *mrp* IE in the ON orientation) [22,30,44]. To complement the phenotype and set the stage for later site-directed mutagenesis experiments, we constructed a new *mrpI* locked-ON mutant with a removable antibiotic resistance marker (*mrpI-*ON), and an *mrpI*-ON *mrpH* double mutant (*mrpI-*ON Δ*mrpH*). The double mutant formed biofilms poorly, comparable to the locked-OFF strain (Fig 7A and 7B). Biofilm formation was restored in the double mutant by complementation with *mrpH* under the control of the native *mrp* promoter on a stable, low copy number plasmid (pGEN-Pmrp-mrpH) (Fig 7B). Because complementation was tested in an *mrpI* mutant, the invertible element in the complementation plasmid always remains in the “ON” orientation.

### Neither tartrate nor glutamic acid inhibit MR/P function

Because we found tartrate and glutamate bound to the Zn^2+^ in MrpH_153_ and MrpH_159_, respectively, (Fig 4 and S2 Fig), we hypothesized that tartrate, glutamate, or a chemically similar molecule might be able to interfere with biofilm formation by acting as a competitive inhibitor. To test this, bacteria were cultured in the presence of 1 mM of L-(+) tartrate or L-glutamate and examined for biofilm formation. However, neither of the tested compounds had any effect on biofilm formation (S5A Fig). Hence, neither of these small-molecule compounds appear to bind tightly to MrpH, and are unlikely to be physiological ligands. We tested additional structurally similar carboxylic acids and amino acids; however, none of these substrates altered biofilm levels (S5A Fig).

We also hypothesized that electrostatic interactions might influence biofilm formation, because of i) the positive charge on the top surface of MrpH, ii) increased biofilm formation at acidic pH, and iii) the general negative charge of polystyrene culture tubes and bacterial lipopolysaccharides. To test this, biofilm cultures were conducted in the presence of increasing concentrations of NaCl (*i*.*e*., increased ionic strength; 10–500 mM). However, this too had no effect on biofilm formation, even at very high concentrations (500 mM NaCl) (S5B Fig).

### Zinc is required for biofilm formation

**Fig 8:**
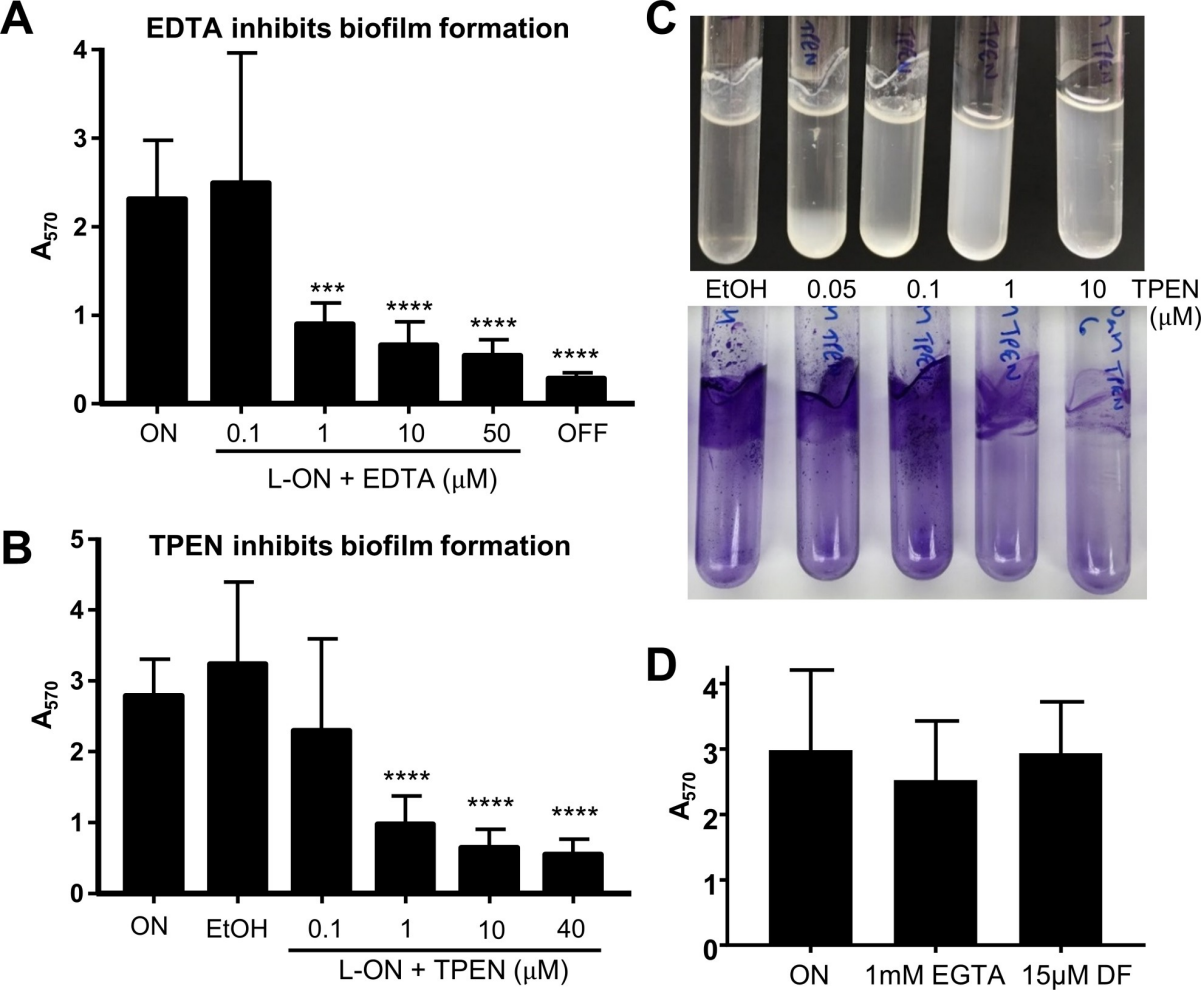
Biofilm formation requires divalent cations. (A) EDTA (n = 4–6) and (B) TPEN (n = 6–8) inhibit biofilm formation. EtOH refers to locked-ON bacteria with ethanol vehicle added. (C) Addition of increasing concentrations of TPEN shifts *P*. *mirabilis* locked-ON from biofilm to planktonic growth. (D) Biofilm formation by *P*. *mirabilis* locked-ON does not differ significantly with chelators EGTA (Ca^2+^-specific) and deferoxamine (DF; iron-specific). ***P < 0.001; ****P < 0.0001 compared to L-ON by one-way ANOVA.

To investigate if divalent cations influence native MrpH-dependent biofilm formation in *P*. *mirabilis*, we studied biofilm formation in the presence of chelators. Addition of divalent metal chelator EDTA to *P*. *mirabilis* L-ON cultures resulted in a strong inhibition of biofilm formation (Fig 8A, S6A Fig). Likewise, TPEN, which is a transition metal chelator with a particularly high affinity for Zn^2+^, also inhibited biofilm formation (Fig 8B, S6B Fig). Increasing concentrations of TPEN resulted in a dose-dependent shift from biofilm to planktonic growth (Fig 8C). In contrast, biofilm formation was not significantly affected in the presence of EGTA, a Ca^2+^-specific chelator, or the iron-specific chelator deferoxamine (Fig 8D). These assays were carried out at chelator concentrations that were not inhibitory for planktonic growth, as seen by the growth of the L-OFF strain in the presence or absence of chelator (S6A and S6B Fig), although it should be noted that the highest concentrations of chelators tested in Fig 8 (50 μM EDTA or 40 μM TPEN) did decrease overall bacterial growth (S6C and S6D Fig). Culture of the L-OFF strain with chelators had no further effect on biofilm formation by that strain (S6E Fig). Hence, MR/P-mediated biofilm formation did not require Ca^2+^ or Fe^2+^ but was dependent on the presence of transition metals/divalent cations. Taken together, this suggests that a transition metal is important for MrpH-dependent biofilm formation, possibly by being required for binding to surfaces or for cross-linking individual *P*. *mirabilis* bacteria in biofilms. Addition of excess Zn to wild-type cultures, however, was not sufficient to increase biofilm formation (S6F Fig).

**Fig 9:**
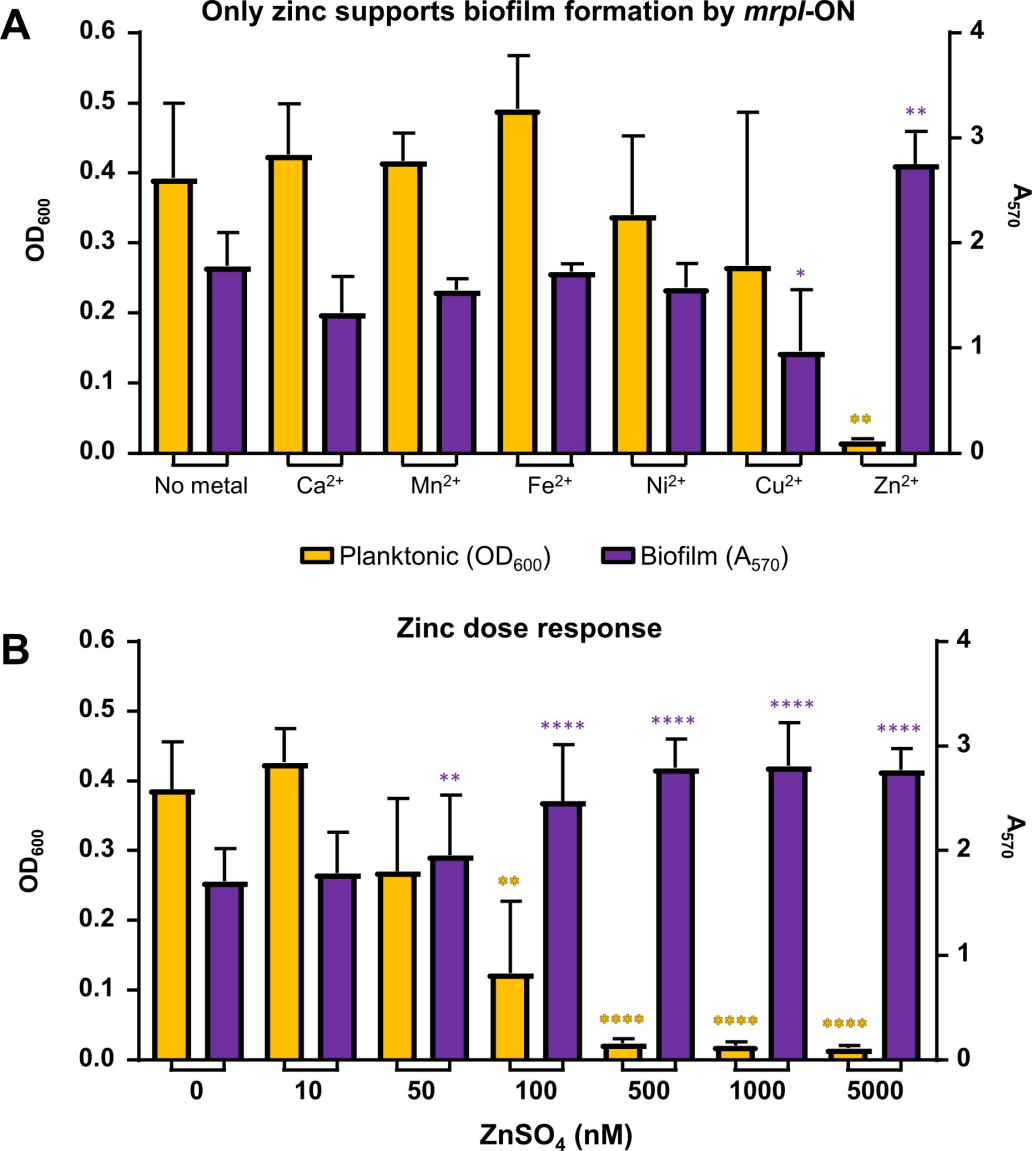
MrpH zinc binding is essential to its function. (A) When *P*. *mirabilis mrpI*-ON was cultured in chelexed medium ± 5 μM metal supplementation, only zinc restored biofilm formation. Significance against “No metal” columns (OD_600_ in orange and A_570_ in purple) calculated by one-way ANOVA with Dunnett’s multiple comparisons test; *P < 0.05; **P < 0.01; n = 3. (B) Biofilm formation is induced by physiologically-relevant levels of zinc. *P*. *mirabilis mrpI*-ON was cultured in chelexed Minimal A + CAA with increasing amounts of ZnSO_4_ for 22 h. Planktonic growth (OD_600_) was recorded, then biofilm was stained and quantified (A_570_). The zinc concentration that induces biofilm formation appears to be 50–100 nM. Significance against 0 nM ZnSO_4_ columns (OD_600_ in orange and A_570_ in purple) calculated by one-way ANOVA with Dunnett’s multiple comparisons test; **P < 0.01; ****P < 0.0001; n = 5.

We then investigated whether Zn^2+^ specifically was required for native MrpH function in *P*. *mirabilis*. To facilitate these experiments, we treated Minimal A medium (pH 6) with Chelex, a filterable chelating agent to remove divalent cations; this rendered *P*. *mirabilis* unable to grow, and only addition of dilute chelated casamino acids restored growth of wild-type *P*. *mirabilis* (S7 Fig). However, this metal-depleted medium resulted in stunted biofilm formation and increased planktonic growth by *mrpI*-ON (S7 Fig). When metal^2+^ solutions were added to this amended medium, only Zn^2+^ restored biofilm formation for *mrpI*-ON (Fig 9A). In a related experiment, an excess of Zn^2+^ added to standard Minimal A pH 6 (*i*.*e*., without casamino acids) was able to overcome biofilm inhibition from TPEN (S7B Fig). Compared with the Chelex-treated experiments, where the chelating agent was removed prior to bacterial culture, TPEN was present throughout the duration of the bacterial culture.

To ascertain the concentration of Zn^2+^ required to support biofilm formation, we measured biofilm formation by *mrpI*-ON in chelexed medium with a range of ZnSO_4_ concentrations. A gradual planktonic-to-biofilm switch was observed from 10–500 nM ZnSO_4_, and addition of ZnSO_4_ up to 5 μM had no further effect (Fig 9B). This range spans the lower end of physiological levels found in urine (while an average labile urinary [Zn] has been reported as 230 nM [45], a more typical range is approximately 2 to 11 μM [46]), indicating that biofilm formation in the urinary tract or on catheters could be dependent on both MR/P fimbriae and zinc levels.

### Zinc-dependent biofilm formation is reversible

**Fig 10:**
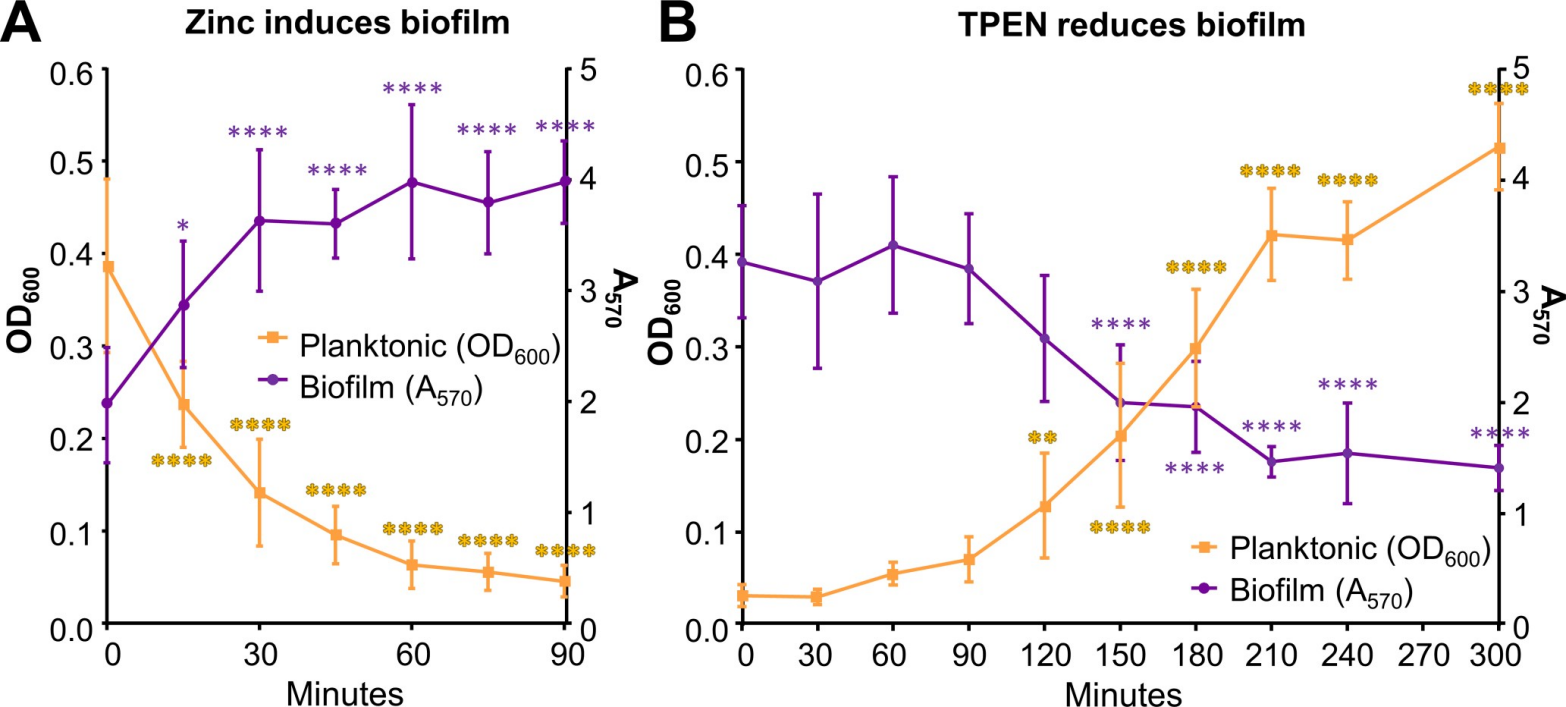
Zinc-dependent biofilm formation is reversible. (A and B) Time course of biofilm reversal. (A) *mrpI*-ON was cultured overnight in chelexed Minimal A + CAA. The next day, 5 μM ZnSO_4_ was added, and cultures were incubated for an additional 90 min. (B) *mrpI*-ON was cultured overnight in chelexed Minimal A + CAA + 5 μM ZnSO_4_. The next day, 10 μM TPEN was added, and cultures were incubated for an additional 3h. Planktonic growth (OD_600_) and biofilm formation (A_570_) were measured in replicate cultures at each time point. n = 5–7. Statistics: one-way ANOVA with Dunnett’s multiple comparisons test, compared with T = 0. *P < 0.05, **P < 0.01, ***P < 0.001, ****P < 0.0001.

The previous experiments showed a clear link between zinc levels and MrpH-dependent biofilm formation, and we wondered if we could use zinc modulation to alter the biofilm phenotypes of stationary phase cultures. To test this, we cultured *mrpI-*ON bacteria overnight in chelexed medium with or without 5 μM ZnSO_4_. To examine the kinetics of reversible biofilm formation, we conducted parallel cultures to measure culture density and conduct biofilm assays. As expected, when zinc was absent, the bacteria primarily grew planktonically (Fig 10A). However, once 5 μM ZnSO_4_ had been added to a planktonic culture in chelexed medium, there was a rapid switch to adherence, with an observable change in 15 min and nearly complete biofilm formation within 60 min (Fig 10A). Likewise, addition of the zinc chelator TPEN to a biofilm-forming zinc-replete culture caused an outgrowth of planktonic bacteria and a significant reduction in biofilm (Fig 10B). The switch from biofilm to planktonic states after TPEN addition was more gradual, occurring over three to five hours.

### Site-directed mutagenesis of selected MrpH residues

**Fig 11:**
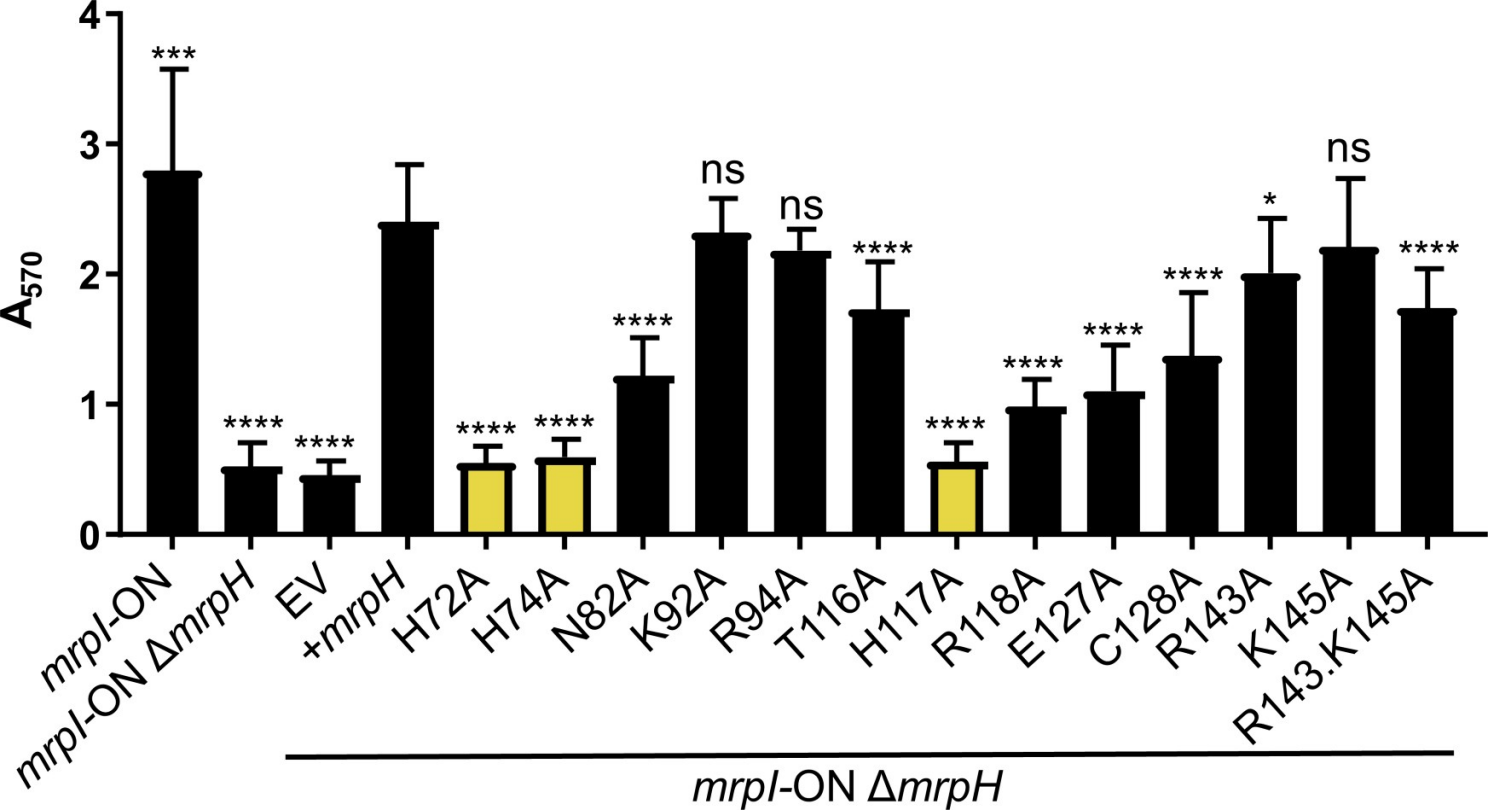
Zinc-coordinating histidines are required for biofilm formation. Biofilm formation by an MR/P locked-ON mutant, a locked-ON *mrpH* double mutant, and the double mutant complemented with wild-type or mutated *mrpH* expressed from plasmid pGEN-Pmrp-*mrpH* (+*mrpH*). pGEN-Pmrp-*luxCDABE* (EV) is a negative control plasmid and has luciferase genes under the control of the native *mrp* promoter. Significance compared against pGEN-Pmrp-*mrpH* using one-way ANOVA with Dunnett’s multiple comparisons test. Compared with the negative (EV) control, these mutants were not significantly different: H72A, H74A, H117A, and R118A; E127A *P* = 0.0130.

To further investigate the importance of the MrpH metal binding site, we carried out site-directed mutagenesis of the Zn^2+^-ligating histidine residues in the complementation plasmid. Each residue (H72, H74, H117; Fig 4) was mutated to alanine. None of these mutants was able to form biofilm (Fig 11), confirming the critical importance of a functional metal binding site for biofilm formation. Construction of double or triple alanine substitutions of these His residues had no further effect on biofilm formation (S8 Fig).

Abrogation of zinc binding by MrpH could lead to MrpH instability and degradation, or MrpH could be assembled as usual at the tip of MR/P fimbriae but unable to function. To distinguish between these possibilities, whole-cell lysates of *mrpI*-ON, *mrpI*-ON Δ*mrpH*, or the double mutant with complementation plasmids were subjected to immunoblot. Although complementation of the double mutant with wild-type *mrpH in trans* restored MrpH levels, when we examined MrpH levels for His-to-Ala mutants expressed *in trans*, very little or no protein was visible (S9 Fig, lanes H72A, H74A, H117A). This might suggest that abrogation of zinc binding does indeed destabilize MrpH. MrpA, however, is much more easily detected compared with MrpH and is demonstrative of the molecular assembly of MrpA with MrpH. If MrpH is not properly folded and secreted, then MrpA will not follow and MrpA will not polymerize into a full fimbrial structure (and MrpA would not be detectable on western blots). Thus, improper folding of MrpH results in greatly diminished MrpA in whole cell lysates. Instead, in immunoblots of MrpH H72A, H74A, or H117A, MrpA levels were comparable to wild-type MrpH expressed *in trans* (S9 Fig). This strongly suggests that the His-to-Ala substitution mutants are producing MrpH that is sufficient to initiate assembly of MrpA into MR/P fimbriae. Therefore, we argue that MrpH is stably produced by the His-to-Ala mutants. We do not know why mutant MrpH is not visible by immunoblot, although one possible explanation is that the antibody was highly affinity purified [23]. The purified antibodies could primarily recognize areas around the zinc binding pocket of MrpH, which is what is altered in the His-to-Ala mutants.

Residues N82, T116, and R118 are close to the metal binding site (S10 Fig) and therefore might contribute to binding a receptor. Alanine substitution of N82 or R118 resulted in a marked decrease in biofilm formation. Specifically, biofilm formation by R118A was not significantly better than the empty vector control, suggesting that this residue together with the Zn^2+^ is part of an extended receptor binding site. The T116A mutant, on the other hand, retained near wild-type levels of function (Fig 11).

Residues E127 and C128 are conserved in MrpH-related adhesins (Fig 3), and C128 takes part in one of two conserved disulfide bonds. Mutation of either residue to alanine resulted in a partial attenuation of biofilm formation (*i*.*e*., the intermediate level of biofilm formation observed was significantly different from both the wild-type complemented strain and the vector control). We also tested whether the highly positively-charged surface of MrpH contributes to its function. K92 and R94 form part of a loop on the opposite side of the top surface to the metal-binding site. R143 and K145 form a positive patch on the midsection of the top surface (S10 Fig). The K92 and R94 residues were dispensable for biofilm formation, as alanine substitution of either one resulted in wild-type levels of biofilm (Fig 11); a similar result was observed for the K145A mutation. Single mutation of R143 or double mutation of R143 and K145 only slightly decreased MrpH function, indicating that these positively charged residues are also not required for MrpH-mediated biofilm formation.

### Hemagglutination is zinc-dependent

**Fig 12:**
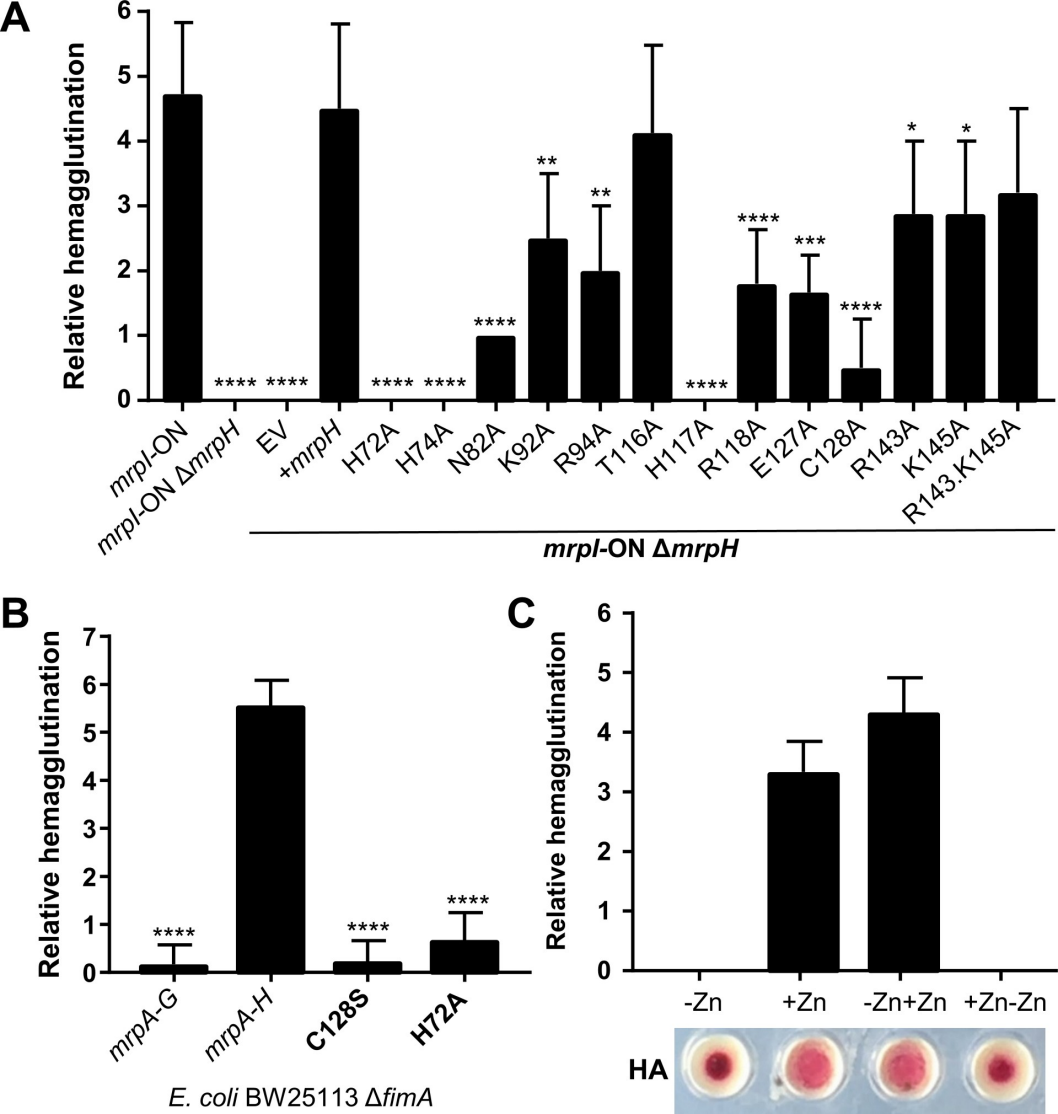
Hemagglutination assays. (A) *P*. *mirabilis* (*mrpI*-ON or *mrpI-*ON Δ*mrpH*) was cultured statically for 48h, washed, then mixed with 3% guinea pig erythrocytes in 2-fold decreasing bacterial dilutions. Hemagglutination (HA) was recorded after 30 min as the last dilution that resulted in HA. Statistical significance calculated against pGEN-Pmrp-*mrpH* complementation strain (+ *mrpH*). (B) HA by afimbriate *E*. *coli* BW25113 Δ*fimA*, cultured as in (A), expressing *mrpA-H* or related mutants from a plasmid. (C) HA mediated by *mrpI*-ON cultured in chelexed medium without or with 5 μM ZnSO_4_ (-Zn or +Zn, respectively). Either 5 μM ZnSO_4_ or 10 μM TPEN was added (-Zn+Zn, or +Zn-Zn, respectively), and after 3 h, bacteria were assayed for HA. Representative HA reactions from the undiluted column of bacteria are shown on the bottom.

Biofilm formation requires interactions both between individual embedded bacteria and, in the assays presented here, with polystyrene or silicone surfaces. However, several lines of evidence indicate that MR/P fimbriae also directly mediate interactions with the mammalian urinary tract, such as by binding to uroepithelial cells and contributing to urinary stone formation [19,30,42]. Indeed, MR/P fimbriae were named for their ability to agglutinate red blood cells in a mannose-resistant manner, further suggesting a mammalian receptor [47]. Therefore, we used hemagglutination (HA) assays to assess the contributions of zinc and site-directed MrpH mutants to adherence to a living substrate. Similar to the biofilm results, mutation of any of the three zinc-binding histidine residues completely abrogated HA (Fig 12A). In general, *mrpH* mutants had similar activity in both assays. An exception is residues K92 and R94, which displayed reduced HA yet retained full biofilm formation ability (Figs 11 and 12A). These two residues form a positively charged surface patch that is on the top surface but distant from the Zn^2+^ site (S10 Fig). Another difference was C128A, which conferred modest biofilm formation but almost no HA (Fig 12A).

We also investigated the effect of producing MR/P fimbriae in an afimbriate *E*. *coli* strain, BW25113 Δ*fimA* [48]. As shown previously, expression of *mrpA-H* in *E*. *coli* resulted in robust HA, while *E*. *coli* expressing *mrpA-G* (missing the *mrpH* adhesin gene) had no HA activity (Fig 12B). In this strain background, the H72A mutation also abrogated HA. Intriguingly, we did not observe biofilm formation when *mrpA-H* was expressed in *E*. *coli* (S11 Fig). This stands in contrast to a previous report, where *E*. *coli* DH5α produced pellicles when *mrpA-H* was expressed [23]. Possible explanations for the different results include the *E*. *coli* strain used and the different conditions for the biofilm assay (22 h aerated or 72 h static culture). However, the prior publication noted that culture tubes needed to be handled carefully to avoid disrupting the pellicle, while we have found that aerated *P*. *mirabilis mrpI*-ON biofilms tenaciously adhere to culture tubes; thus, it may be that *P*. *mirabilis* contributes additional elements to biofilm formation that are missing in these *E*. *coli* strains. We also found that wild-type MrpH was undetectable by immunoblot when expressed as part of the entire set of *mrp* structural genes (*mrpA-H*) in *E*. *coli* (S9 Fig). Interestingly, MrpA was also barely detectable in this *E*. *coli* background, yet the amount of protein produced was sufficient to induce MR/P-dependent hemagglutination (Fig 12B).

To test the contribution of zinc to HA, we conducted HA assays on *P*. *mirabilis mrpI*-ON that had been cultured in aerated chelexed minimal medium, as for Fig 10. Similar to the biofilm results, HA only occurred when ZnSO_4_ was included in the culture. This phenotype was also reversible, as addition of ZnSO_4_ to zinc-free chelexed medium restored HA. Likewise, addition of TPEN to a zinc-replete culture eliminated HA (Fig 12C). Because tartrate was bound to Zn^2+^ in MrpH_153_, we also tested whether 50 mM tartrate could act as a competitive inhibitor of HA. However, similar to the biofilm assays, tartrate had no effect on HA by *mrpI-*ON.

### Zinc binding likely contributes to UTI

A limitation of the prior experiments is a reliance on *mrp* IE-locked strains. In general, *mrp* genes are not expressed well under laboratory conditions, and the IE is typically in the OFF orientation. However, during experimental UTI, the switch is overwhelmingly ON, removing the necessity of locking the switch to observe MR/P-dependent phenotypes [29,44]. Furthermore, MR/P fimbriae are essential contributors to pathogenesis in a mouse model of UTI [9]. To test whether the zinc-binding site contributes to virulence, we tested the H72A mutant in ascending UTI. Specifically, we did not use *mrpI* mutants in the mouse UTI model experiments, meaning that the invertible element controlling the *mrp* promoter was free to switch ON and OFF.

**Fig 13:**
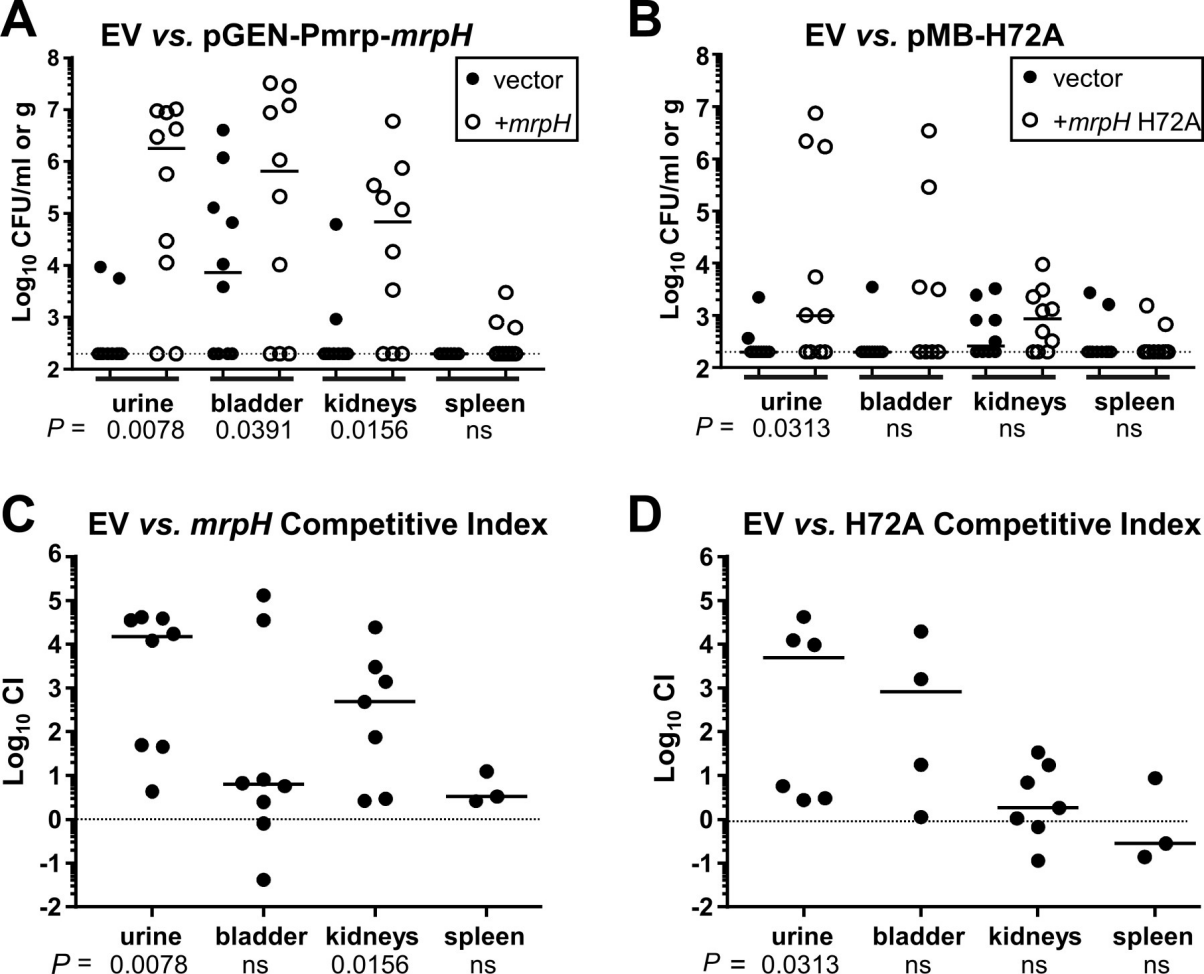
Zinc-coordinating residue H72 contributes to UTI. Bacterial recovery from female CBA/J mice at 7 dpi. *P*. *mirabilis* HI4320 *mrpH*Δkan (kan^S^) containing the empty vector pGEN-MCS (EV) was competed in a 1:1 ratio with HI4320 *mrpH*Ωkan (kan^R^) containing either wild-type *mrpH* (pGEN-Pmrp-*mrpH*) or *mrpH* H72A (pMB-H72A). (A and B) CFU recovery of *P*. *mirabilis* from urine or organs. Bar indicates median CFU. Limit of detection = 200 CFU, indicated with a dotted line. (C and D) Competitive indices (CI) of the experiments shown in A and B, showing the ratio of recovered (output) to input vector:complemented bacteria. The dotted line at *y* = 0 indicates no competitive advantage for either strain. Log CI > 0 indicates that the *mrpH*-complemented bacteria outcompeted the vector control. ns, not significant.

When a *P*. *mirabilis mrpH* mutant containing an empty vector was mixed with the same mutant containing *mrpH* complementation vector pGEN-Pmrp-*mrpH* and co-inoculated into mice, the *mrpH*-expressing strain was recovered much more readily from the urine, bladders, and kidneys after a seven-day infection (Fig 13A and 13C). In contrast, the H72A mutant fared poorly, and was recovered in numbers comparable to the vector control from the bladders, kidneys, and spleens; the mutant was recovered in slightly higher numbers from the urine of infected mice, suggesting that the mutant retained a low level of function (Fig 13B and 13D). Fig 13A and 13B show the absolute quantity of CFU recovered from each site, while Fig 13C and 13D show competitive indices, that is, the ratio of *mrpH*-complemented bacteria to the vector control obtained from each mouse.

Notably, when wild-type *mrpH* was expressed *in trans*, we did not observe urinary stone formation or high levels of bladder inflammation that are common for *P*. *mirabilis* in mouse model experiments; this may be due to dysfunctional MR/P fimbrial production or assembly when *mrpH* is expressed from a plasmid, or, more likely, because the chromosomal *mrpH* mutation is polar on *mrpJ*. MrpJ is a transcriptional regulator that controls levels of multiple known and predicted virulence factors, including flagella, MR/P and other fimbriae, and a type VI secretion system [28,49]. Further study will be needed to tease apart the separate roles of MrpH and MrpJ during UTI.

## Discussion

In this study, we discovered that MrpH_ntd_ has a distinctive structure and metal-binding capability, making this fimbrial protein stand out from other chaperone-usher adhesins. The Zn^2+^-binding site, consisting of three conserved histidine side chains, resembles the classical zinc binding site of α-, γ- and δ-carbonic anhydrases. We also discovered that an intact Zn^2+^-binding site occupied by Zn^2+^ was required both for biofilm formation and for hemagglutination, and that biofilm formation could be reversed by metal depletion using transition metal chelation.

The zinc dependence of MrpH-mediated biofilm formation could be caused either by zinc being required for direct receptor interactions, or for MR/P fimbrial assembly on the bacterial cell surface. Addition of zinc to chelated planktonic cultures led to rapid initiation of biofilm formation (<15 min) (Fig 10A), strongly suggesting that MR/P fimbriae are assembled also when zinc is scarce, and exogenously-supplied zinc can activate binding to MrpH targets. Mutations of the Zn^2+^-interacting histidine residues completely eliminated both MrpH-mediated biofilm formation and HA.

While it is possible that mutation of these histidine residues leads to protein degradation or conformational changes that prevent correct fimbrial assembly, our data indicate that this is not the case. The histidine sidechains are surface exposed and have no obvious structural role in stabilizing the fold. Direct detection of MrpH is difficult; in MR/P fimbriae, a single copy of the MrpH adhesin is displayed at the tip of a fimbrial rod made up of ~1000 MrpA subunits. All chaperone-usher pathway fimbriae are assembled tip-to-base, and when a TDA such as MrpH is part of the structure, assembly requires the secretion of a correctly folded adhesin. In the best-characterized examples, *E*. *coli* type 1 and P fimbriae, there is ample evidence that the adhesin is the first subunit incorporated into the fimbria and is required for initiating fimbrial assembly by triggering usher activation [50–54]. Although details of the MR/P fimbriae assembly process are not as well studied, deletion of the MrpH tip adhesin abolishes MR/P fimbriation [23]. Hence, detection of the major fimbrial subunit MrpA can be used to assess the presence of a stably folded and secreted MrpH. Immunoblots of MrpH and MrpA suggest that the mutations do not abrogate fimbrial assembly and hence do not significantly affect the fold or stability of MrpH (S9 Fig). Therefore, we conclude that the loss of function in MrpH with a mutated zinc binding pocket is likely caused by loss of zinc binding.

### Zinc-dependent biofilm formation is a target for therapeutic intervention

We found that zinc is required for MR/P-dependent biofilm formation, and this occurs at concentrations of zinc that overlap with the physiological range found in human urine (Fig 9). This suggests that *P*. *mirabilis* biofilm formation in the urinary tract or on catheters may depend on urinary zinc levels. Zinc is an important contributor to host innate immune responses [55], and the host restricts available zinc for invading pathogens during infection [56]. Relatively little is known about modulation of urinary zinc during infection; however, neutrophils, which sequester zinc via production of calprotectin, are a frontline innate defense for UTI [4,57,58]. Furthermore, multiple studies have pointed to the importance of zinc for uropathogenesis [59–61]. Urinary zinc levels are also responsive to host diet and specific disease states [46,62]. High doses of zinc have been linked to increased risk of UTI and urolithiasis [63], a finding that is particularly relevant for stone-forming *P*. *mirabilis*.

Zinc homeostasis contributes to *P*. *mirabilis* fitness during UTI [64]. We now show that transition metal chelators inhibit biofilm formation (Fig 8). More importantly, existing biofilm can be reverted to planktonic growth after addition of the zinc chelator TPEN (Fig 10). Collectively, this points to a possible translational application for control of *P*. *mirabilis* catheter biofilms via manipulation of urinary zinc levels, or development of anti-biofilm catheter materials or coatings. Indeed, the general divalent cation chelator EDTA was previously shown to interfere with *P*. *mirabilis*-mediated encrustation of urinary catheters in an *in vitro* setting [65]. Zinc chelation also inhibits biofilm formation by staphylococci, suggesting that zinc chelation shows broad promise for combatting catheter-associated infections [66].

A hallmark of *P*. *mirabilis* UTI is basic urinary pH as a consequence of urease activity. Therefore, the finding that MrpH-dependent biofilm formation is enhanced at acidic pH seems paradoxical. One explanation is increased thermal stability of MrpH at lower pH. Healthy urine is normally slightly acidic, and this could facilitate early colonization events by *P*. *mirabilis*. The *mrp* operon is highly induced at early stages of experimental infection (24 hpi), and transcription tapers off over the course of 7 days [22]. Thus, MR/P fimbriae might be most effective during the initial stages of infection, prior to urease-mediated alkalization. With this knowledge, we suggest that interference with MR/P-mediated biofilm formation may be most beneficial as an early intervention. Identification of MrpH receptors will be key to answering these questions.

When *P*. *mirabilis* colonizes urinary catheters during CAUTI, urease-dependent crystalline biofilms form, where bacteria are encased in struvite and apatite crystals [18]. MR/P-dependent biofilm formation does not require urease *in vitro*, but importantly, biofilm architecture appears to be determined by both urease-mediated mineralization and MR/P fimbriae [19,30]. Urease-mediated crystalline biofilm formation is such a pervasive problem in catheter blockage [67] that it seems likely that successful anti-biofilm interventions will need to target both urease and fimbrial adherence.

### Does the hammerhead orientation of MrpH alter its binding properties?

All previously determined fimbrial NTD structures share the same immunoglobulin-like fold, with 7 β-strands arranged in a β-sandwich structure [34] (Fig 2B). The MrpH NTD has a fold that is distantly related to but distinct from this canonical fold, with β-strands E-B-A-G and F-C-D making up the two β-sheets of the sandwich (Fig 2A), in contrast to canonical TDAs where one sheet is made from strands B-E-D and the second from strands A-G-F-C. In canonical TDAs, the N-terminus is located close to the tip of the NTD, and the final strand, G, stretches along the whole domain and connects to the TDA pilin domain via a linker at the opposite side of the domain (Fig 2B), resulting in a more or less in-line orientation of the receptor-binding NTD with respect to the fimbrial axis. In contrast, the MrpH_ntd_ G strand ends at a conserved proline-rich segment near the middle of the domain (Figs 2A and 3), suggesting a hammerhead orientation with respect to the fimbrial axis.

The type 1 fimbrial adhesin FimH demonstrates “catch-bond” binding kinetics, where shear force converts the NTD from a weak-binding to a tight-binding conformation [68–70]. If, as suggested by the structure, the NTD β-sheets in MrpH are arranged more or less perpendicular to the MR/P fimbrial axis, in contrast to the parallel orientation in FimH, shear force would not be able to propagate in the same way in MrpH to get the relative twist of the two NTD β-sheets affecting the binding site in FimH. However, MR/P-dependent biofilm formation was most readily observed in aerated cultures, where shear force would be in play. Our lack of success thus far in finding an MrpH_ntd_ ligand could be because it is in a weak-binding conformation, and so it will be informative to obtain additional MrpH structures, *e*.*g*., a structure for full-length MrpH, either in complex with its chaperone or as a self-complemented construct.

### MrpH-like adhesins in other species

MR/P fimbriae are ubiquitous in *P*. *mirabilis* [10], and BLAST of MrpH shows that homologs are widespread in related species of *Morganellaceae* (recently reclassified from *Enterobacteriaceae*) [37,71]. Thus, zinc-dependent adherence may be a common theme for *Proteus*, *Providencia*, and *Morganella* genera. These three genera all contain species that are common in patients undergoing long-term urinary catheterization, although the role of MR/P fimbriae during UTI has thus far only been assessed for *P*. *mirabilis* [10]. Little is known about MrpH homologs outside of *P*. *mirabilis*, but two examples indicate they contribute to other host-bacterial interactions: the related Mrx fimbriae of *Xenorhabdus nematophila* contribute to mutualistic colonization of nematodes [72], and genes encoding Mrf fimbriae of *Photorhabdus temperata* are expressed during later stages of experimental insect infection [73]. Both of these species are also classified as *Morganellaceae* [71]. BLAST analysis also reveals many examples of MrpH-related two-domain adhesins in other members of the *Enterobacterales*, particularly in *Serratia spp*., where it remains uncharacterized. Each of these examples includes the metal-binding histidine residues, four conserved cysteines that participate in disulfide bond formation, and conserved prolines that contribute to the hammerhead conformation. The presence of MrpH-like fimbrial adhesins in other pathogenic species suggests that any therapeutic intervention targeting MrpH developed for *P*. *mirabilis* UTI might be more generally applicable.

### What does MrpH bind?

While the MrpH receptor remains unknown, how MR/P fimbriae drive bacteria to form biofilm communities on polystyrene culture tubes or silicone urinary catheters could proceed by several different scenarios. Biofilm formation on abiotic surfaces will typically depend on establishment of interactions of bacteria with the surface as well as between themselves. MrpH might be involved in both sets of interactions, either by direct binding to receptors on the abiotic surface and/or the *P*. *mirabilis* cell surface, or via binding to something in the culture milieu or secreted by *P*. *mirabilis*. It is intriguing that MR/P fimbriae confer robust biofilm formation on *P*. *mirabilis* but not *E*. *coli*, even though expression of these fimbriae in both species results in HA activity. This suggests that *P*. *mirabilis* contributes an additional component to MR/P-dependent biofilm formation, and we speculate that MrpH might bind something on the *P*. *mirabilis* surface, thereby causing the bacteria to interact with each other and establish a biofilm community. Alternatively, a bi- or multi-functional biofilm matrix component specific to *P*. *mirabilis* biofilm might serve to crosslink bacteria by allowing binding of two or more MrpH adhesin molecules at the same time.

Possible physiological ligands that could function as receptors would include acidic side chains of proteins or sialoglycans. The serendipitous finding of two different carboxyl acid ligands providing thermal stabilization (increased T_m_ in TSA) and binding to MrpH_ntd_ in the same location and in a similar manner in our two independent crystal structures suggests that the ligand-binding site may be used for physiological receptor binding and provides some hints as to the nature of a receptor. The ligand-binding site together with the crevice extending from the ligand-binding site across the positively charged top surface of MrpH_ntd_ might allow MrpH to accommodate an extended ligand or receptor with a carboxyl group for binding to the Zn^2+^ in the metal binding site. It is interesting to note that MrpH_ntd_ was thermally more stable in the presence of glutamate, but not aspartate, glutamine, or asparagine, indicating some level of specific binding to the glutamic acid side chain. Since neither glutamate nor tartrate inhibited biofilm formation, these molecules do not appear to bind tightly to MrpH. However, given the possibility of an extended MrpH receptor-binding site, it is tempting to speculate that the receptor is a more complex structure with multiple binding sites, such as a protein with a surface-located glutamic acid side chain binding to the Zn^2+^. If this is the case, the absence of any inhibitory effect of tartrate or glutamate might not be entirely surprising. Protein-protein interfaces are typically large and relatively flat, and therefore difficult to disrupt with small molecules [74,75]. In addition, an inhibitor would have to overcome the significant avidity effect expected from multivalent binding of *P*. *mirabilis* cells covered in MR/P fimbriae.

While most known receptors for chaperone-usher fimbriae are carbohydrate moieties attached to proteins or lipids, several examples of non-carbohydrate receptors have been described. For example, the enterotoxigenic *E*. *coli* colonization factor CS6 binds to fibronectin [76], *E*. *coli* Dr-family adhesins bind to non-glycosylated parts of carcinoembryonic antigen [77], and *Acinetobacter baumannii* Csu fimbriae mediate biofilm formation by binding to hydrophobic surfaces on plastic [78]. No experiments thus far have pointed toward a carbohydrate receptor for MrpH. Notably, the observation of mannose resistance that was used to name this fimbria merely indicated that mannose was not a receptor [47,79].

We found that some site-directed mutants had differing phenotypes in HA and biofilm assays. For example, K92 and R94 were dispensable for biofilm formation but had a modest contribution to HA titers. On the other hand, T116A retained full HA activity but was slightly reduced in biofilm formation. This may point toward different MrpH targets in the host compared with biofilm formation on abiotic surfaces. We note that a C128S mutation expressed in *E*. *coli* was previously reported to eliminate both biofilm formation and HA [23]. Although we found that C128A HA activity was greatly reduced, we found only a partial attenuation of biofilm formation by the C128A mutant. However, the prior report found that MR/P-dependent *E*. *coli* biofilms were easily dispersed, and therefore intermediate biofilm phenotypes may be easier to observe in a robust biofilm-forming *P*. *mirabilis mrp*-ON background. Studies to identify the physiological MrpH receptor in the urinary tract are under way.

In summary, our studies revealed that the MrpH structure has a unique fold and contains a Zn^2+^ binding site, something that has not, to our knowledge, been previously reported for any fimbrial adhesin. An intact metal binding site is required for biofilm formation in culture tubes and catheter segments, suggesting that it is involved in binding to *P*. *mirabilis* cell surface receptors or biofilm matrix components, perhaps by cross-linking bacteria, or possibly binding to abiotic surfaces. At the same time, zinc binding is required to agglutinate red blood cells, suggesting a specific host receptor. Identification of the MrpH receptor(s) and development of methods to mitigate catheter-associated biofilm formation remain a crucial area for further research.

## Materials and methods

### Bacterial strains and media

*P*. *mirabilis* HI4320 was isolated from the urine of a long-term catheterized patient in a nursing home [6]. Mutants that constitutively produce MR/P fimbriae, “locked ON” (L-ON), or do not produce MR/P fimbriae, L-OFF, were generated by mutation of *mrpI*, which encodes a recombinase that controls an invertible element in the *mrp* operon promoter, and have been previously described [20]. A second MR/P locked ON mutant, *mrpI-*ON, was generated as described below, and was used for site-directed mutagenesis studies. *E*. *coli* K12 or B derivatives were routinely used for construction and maintenance of plasmids. For production of MR/P fimbriae in *E*. *coli*, afimbriate strain BW25113Δ*fimA* was obtained from the Keio collection [48]. A comprehensive list of all strains used in this study is provided in S2 Table.

Bacteria were routinely cultured in lysogeny broth (LB; per liter, 10 g of tryptone, 5 g of yeast extract, and 0.5 g of NaCl) or on LB solidified with 1.5% agar. A chemically-defined medium, Minimal A [43], was adjusted to pH 6 before use in biofilm experiments. When required for plasmid maintenance or mutant selection, 100 μg/ml of ampicillin or 25 μg/ml of kanamycin was added to media. To remove polyvalent metal ions for metal limitation studies, Minimal A was prepared without a carbon source, then slowly stirred with 1 g/L of Chelex 100 resin (Bio-Rad) for at least 15 h. Glycerol was then added, and chelex treatment continued for an additional 3 h. Chelexed medium was filter-sterilized, which also served to remove chelex beads. Separately, a 2% solution of casamino acids was chelexed twice for 3 h, filter-sterilized after each treatment, and added to chelexed Minimal A medium at a final concentration of 0.0002%.

### Construct design and cloning for crystallography

A construct encoding MrpH residues 1–159 (wuMrpHm) was initially designed and used to obtain MrpH_159_ protein for crystallization. cDNA coding for MrpH (PMI0270) from *P*. *mirabilis* wild-type strain HI4320 (codon optimized for *E*. *coli* expression) was purchased from GenScript. The construct was PCR-amplified using Phusion DNA polymerase (ThermoFisher Scientific). The purified *mrpH* DNA fragment was TOPO-cloned into pEXP5-CT/TOPO, transformed into *E*. *coli* Top 10 cells (Invitrogen) and verified by sequencing. *E*. *coli* BL21-AI, BL21-STAR, Origami, and C43 were transformed with recombinant plasmids for protein production trials, and successful expression confirmed by western blot analysis using anti His-tag antibody (monoclonal, Sigma-Aldrich). Based on the expression levels, *E*. *coli* BL21-AI cells containing the cloned plasmid were used for further protein production.

Additional constructs encoding the MrpH N-terminal domain (MrpH_ntd_) were obtained from the Protein Science Facility (PSF), Karolinska Institute, Stockholm, Sweden (http://ki.se/en/mbb/protein-science-facility) as previously described for AtfE and UcaD NTDs [33]. Results of the PSF construct screening are shown in S1 Table. The construct encoding MrpH residues 1–153 (psfMRPH-c001) was used to produce MrpH_153_ protein for crystallization.

### Protein expression and purification

For production of MrpH_153_ or MrpH_159_, bacteria were cultured at 37°C in 8 L of LB containing 50 mg/ml kanamycin or 100 mg/ml ampicillin, respectively, to an OD_600_ of 0.5–1.0. Expression of the target gene was induced by adding IPTG (MrpH_153_) or L-arabinose (MrpH_159_) to final concentrations of 0.5 mM and 0.2% w/v, respectively. After 20 h, the cells were harvested by centrifugation, and the cell pellet was resuspended in lysis buffer (20 mM HEPES, pH 7.5, 300 mM NaCl, 20 mM imidazole, 0.5 mM tris(2-carboxyethyl) phosphine (TCEP), 5% glycerol, one tablet of cOmplete protease inhibitor cocktail (Roche), 10 μg/ml RNase A (Sigma-Aldrich) and 20 μg/ml DNase (from bovine pancreas, Sigma-Aldrich), and lysed using a One Shot cell disruptor (Constant Systems Ltd., UK). The soluble fraction was incubated with 1.5 ml pre-equilibrated Ni-nitrilotriacetic acid agarose (Qiagen) slurry for 1 h at 4°C. The resin was then washed with 20 column volumes of 50 mM imidazole in the same buffer, and the protein eluted with six column volumes of 500 mM imidazole in the same buffer. The eluted fractions containing the desired protein were pooled and further purified on a HiLoad 16/60 Superdex 75 column (GE Healthcare) pre-equilibrated with 20 mM HEPES, pH 7.5, 300 mM NaCl, and 5% glycerol. The eluted fractions were concentrated using a 5 kDa cutoff Vivaspin concentrator (Vivascience). The final concentrated protein samples were analyzed by SDS and native PAGE to assess purity and homogeneity, and stored in 20 mM HEPES buffer pH 7.5 with 300 mM NaCl at -20°C. Prior to use, the protein samples were transferred to 10 mM HEPES pH 7.5, 100 mM NaCl, 5% glycerol.

### Thermal shift assay

Thermal shift assays (TSA) were carried out with different buffers covering a range of pH values and additives using a protocol adapted from Boivin *et al* [40]. Each 25 μl sample consisted of 10 μM purified MrpH_159_ protein, 10 mM ligand or buffer (except for aspartic acid; 4 mM), 5x SYPRO Orange dye, and 24 mM NaCl, 6 mM HEPES, pH 7.5. Measurements were performed in triplicate using a Bio-Rad CFX Connect real-time system and subjected to a temperature gradient from 15 to 95°C with an increment of 1°C/30 s. Raw fluorescence data over the measured temperature *(T)* range was normalized *(f)*, and then further analyzed by non-exponential curve-fitting (GraphPad Prism 8) using the sigmoidal Boltzmann equation

$$f(T)=fmin+\frac{fmax-fmin}{1+{exp}^{\frac{Tm-T}{a}}}$$

to yield the protein melting temperature *(T*_*m*_*)* for each reaction.

### Crystallization

Crystallization was carried out by sitting-drop vapor diffusion using a Mosquito Crystal crystallization robot (TTP Labtech, England). Screening for MrpH_159_ crystallization conditions was carried out by mixing equal volumes (0.2 μl) of reservoir solution and 23.6 mg/ml MrpH_159_. We initially obtained diffraction quality crystals at 20°C in Morpheus HT screen (Molecular Dimensions, UK) condition D9 (0.02 M 1,6-hexanediol, 0.02 M 1-butanol, 0.02 M 1,2-propanediol, 0.02 M 2-propanol, 0.02 M 1,4-butanediol, 0.02 M 1,3-propanediol, 0.1 M bicine/Trizma base pH 8.5, 10% w/v PEG 20000 and 20% v/v PEG MME 550); however, despite repeated trials, we were unable to reproduce the initial MrpH_159_ crystals. Crystallization trials with MrpH_153_ (21 mg/ml) protein in ammonium tartrate buffer produced thin plate-like crystals in Morpheus HT screen (Molecular Dimensions, UK) condition C9 (0.03 M sodium nitrate, 0.03 M sodium phosphate dibasic and 0.03 M ammonium sulfate, 0.1 bicine/Trizma base pH 8.5, 10% w/v PEG 20000 and 20% v/v PEG MME 550). These MrpH_153_ crystals could be reproduced. For single anomalous dispersion (SAD) data collection, MrpH_153_ crystals were soaked in K_2_PtCl_4_ for one day and then transferred to crystallization solution without K_2_PtCl_4_ before flash-freezing in liquid nitrogen.

### Data collection and structure determination

All data were collected at 100 K at the European Synchrotron Radiation Facility (ESRF), Grenoble, France, from one single crystal per dataset. MrpH_153_ SAD data to 1.26 Å were collected at beamline ID23-2, from one Pt soaked MrpH_153_ crystal. Native MrpH_153_ data to 1.02 Å and native MrpH_159_ data to 1.75 Å were collected at beamline ID23-1. Crystals of MrpH_153_ belonged to space group P2_1_ with one molecule in the asymmetric unit. The MrpH_159_ crystals belonged to space group P2_1_2_1_2_1_, also with one protomer in the asymmetric unit.

Diffraction data were processed using *XDS* [80] and scaled and merged using the *CCP4* [81] program *SCALA* [82]. The structure of MrpH_153_ was solved by experimental phasing using the Pt SAD data and completed and refined against the 1.02 Å native data. Experimental phasing and initial automatic model building was done with *SHELX C/D/E* [83] followed by manual building in *Coot* [84] and *O* [85] starting from a few residues autobuilt by *SHELX E* [83]. MrpH_159_ was solved by molecular replacement using *Phaser-MR* [86], with the MrpH_153_ structure as the search model, and completed and refined against the 1.75 Å data. Refinement was carried out using *Refmac5* [87] and the *Phenix*.*refine* module [88] of *Phenix* [89]. MrpH_159_ was modelled with isotropic temperature factors and TLS, whereas anisotropic temperature factors were used for MrpH_153_. Both models were completed using *Coot* [84]. Structure validation was carried out using the tools available in *Phenix*.*refine* [88] and *Coot* [84]. Resolution cut-offs were based on CC½ [90] and completeness of the data. Native data collection and refinement statistics are listed in Table 1. SAD data collection and phasing statistics are shown in S3 Table.

### Structure and amino acid sequence analyses and representation

Structures were superimposed using *Chimera MatchMaker* [91]. *BLAST* [37] was used to identify MrpH_ntd_ homologs in the non-redundant protein database using default settings. Multiple sequence alignment of representative sequences selected in sequence similarity intervals ranging from 100% to 29% (E-values from 9x10^-114^ to 5x10^-07^) was performed with *COBALT* [92] and visualized using *ESPript 3*.*0* [93]. Graphical representations of protein structures were prepared using *UCSF Chimera* [94].

### Electroparamagnetic resonance

A sample of 250 μl native MrpH_159_ protein (1 mM) in 200 mM NaCl, 25 mM Tris, pH 7.0, 3% glycerol was transferred into an EPR tube and immersed in an ethanol-dry ice bath and stored in liquid nitrogen prior to EPR examination. The EPR spectrum was recorded on a Bruker EMXmicro spectrometer equipped with an EMX-Primium bridge and an ER4119HS resonator with temperature controlled using an Oxford Instruments ESR 900 flow cryostat. The EPR recording settings were: microwave frequency 9.38 GHz; microwave power 0.126 mW; temperature 20 K; modulation frequency 100 kHz; modulation amplitude 10 G.

### Total Reflection X-ray Fluorescence (TXRF)

The metal content of purified native MrpH_159_ protein (0.8 mM) and its buffer solution (200 mM NaCl, 25 mM Tris, pH 7.0, 3% glycerol) was quantified using TXRF on a Bruker PicoFox S2 instrument. Measurements were carried out on three independently prepared samples. A gallium internal standard at 2 mg/ml was added to the samples (v/v 1:1) before the measurements. TXRF spectra were analyzed using the software provided with the spectrometer.

### Biofilm formation assays

Overnight LB cultures of *P*. *mirabilis* HI4320 wild type, locked ON, or locked OFF were diluted 1:100 into 2 ml of Minimal A medium in 5 ml culture tubes and incubated in a shaker at 37°C overnight (22 h). Except where specified, Minimal A medium was adjusted to pH 6 to induce biofilm formation. The next morning, cultures were examined for turbidity and biofilm formation, and when applicable, optical density at 600 nm (OD_600_) was measured to assess planktonic growth. Biofilms were stained with 0.1% crystal violet, washed with water three times, then photographically documented. To quantify biofilm formation, 2 ml of ethanol was added for 15 min in a shaker incubator to extract the crystal violet. A 200 μl aliquot of extract was transferred to a 96-well plate and the absorbance at 570 nm (A_570_) measured using a μQuant spectrophotometer (BioTek). Biofilm data were plotted and analyzed using GraphPad Prism 7 software.

When specified, Minimal A medium was supplemented with different metal chelators [2,2′,2″,2‴-(ethane-1,2-diyldinitrilo)tetraacetic acid (EDTA), N,N,N′,N′-tetrakis(2-pyridinylmethyl)-1,2-ethanediamine (TPEN), ethylene glycol-bis(β-aminoethyl ether)-N,N,N′,N′-tetraacetic acid (EGTA), and deferoxamine]. In other biofilm assays, to explore possible MrpH ligands, sodium L-(+)-tartrate dihydrate, L-glutamic acid, L-glutamine, L-aspartic acid, or L-asparagine was added in the specified concentrations. To assess ionic effects on biofilm formation, NaCl was added to biofilm assays at concentrations of 10–500 mM. Metal complementation biofilm assays were performed by adding 5 μM of CaCl_2_, MnSO_4_, FeSO_4_ · 7H_2_O, NiSO_4_ · 6H_2_O, CuSO_4_ · 5H_2_O, or ZnSO_4_ · 7H_2_O to chelexed Minimal A. ZnSO_4_ was also tested at a variety of concentrations (10 nM– 5 μM) with chelexed Minimal A.

To examine the effect of zinc or TPEN on biofilms over time, *P*. *mirabilis mrpI*-ON was cultured overnight in LB, then diluted 1:100 and cultured for 22 h in chelexed Minimal A medium with or without 5 μM ZnSO_4_. The next day, 10 μM TPEN was added to replicate zinc-containing cultures, or 5 μM ZnSO_4_ was added to replicate zinc-free cultures. Replicate cultures were assessed for planktonic (OD_600_) and biofilm (A_570_) populations at specified time points using the staining method outlined above.

Biofilm formation on urinary catheters was assessed by cutting a silicone Foley catheter (16 Fr, 10 ml; Medline) into 5 cm segments, which were placed into 15 ml polystyrene culture tubes filled with 2 ml of Minimal A. Bacteria were cultured for 22 h at 37°C with aeration. Catheter segments were stained with crystal violet, transferred to new culture tubes, and biofilm formation was quantified as above.

### Growth curve analysis

To assess growth in planktonic culture over time, overnight cultures of bacteria were diluted 1:100 into relevant culture media and aliquoted in triplicate into 100-well plates. Plates were placed into a Bioscreen C (Growth Curves USA) set to 37°C with continuous shaking, and OD_600_ was recorded every 15 min for the duration of the experiment.

### Generation of *mrpI-ON* and *mrpH* mutants

*mrpI*-ON and *mrpI*-ON *mrpH* mutants were constructed using a modification of the TargeTron Gene Knockout System (Sigma) as previously described [95,96]. Briefly, a plasmid containing a targetron targeting *mrpI*, pANN128, was used to mutate *P*. *mirabilis* HI4320 that had been cultured under microaerobic conditions to increase the proportion of IE-ON bacteria. Resulting kanamycin-resistant mutants were screened for the orientation of the *mrp* promoter invertible element using a PCR-based assay [44], and a mutant with the invertible element in the ON orientation was selected (*mrpI*-ON). The kanamycin resistance cassette was removed from *mrpI*-ON using *cre*/lox recombination as previously described [97], generating a markerless mutant. Then, *mrpH* was mutated using a plasmid with an *mrpH*-specific targetron (pANN126) to generate the *mrpI-*ON *mrpH* double mutant. Primers used for targetron mutagenesis and the resulting plasmids are listed in S2 and S4 Tables.

Plasmid pANN126 was also used to mutate *mrpH* in wild-type *P*. *mirabilis* HI4320, creating HI4320 *mrpH*Ωkan. The kanamycin resistance cassette was subsequently removed, creating HI4320 *mrpH*Δkan.

### *mrpH* plasmid complementation and site-directed mutagenesis

To complement the *mrpI-*ON *mrpH* mutant, *mrpH* was cloned into the stable, low copy number plasmid pGEN-MCS [98] under the control of the native *mrp* operon promoter to create plasmid pGEN-Pmrp-*mrpH*. In detail, the *mrp* operon promoter, extending from *mrpI* to *mrpA*, was amplified by PCR from HI4320 chromosomal DNA and cloned in front of promoterless luciferase genes in pGEN-*luxCDABE* [98] using KpnI and BamHI restriction sites, resulting in pGEN-Pmrp-*luxCDABE*. Next, *mrpH* was PCR amplified from HI4320 chromosomal DNA. Following digestion with BamHI and SalI, the luciferase genes were removed from pGEN-*luxCDABE*, and *mrpH* inserted after the *mrp* promoter, resulting in pGEN-Pmrp-*mrpH*. To construct site-directed mutations, DNA primers corresponding to the target *mrpH* sequence were designed, with nucleotides altered to encode alanine codons in place of histidine (S4 Table). These primers were used in a PCR reaction with pGEN-Pmrp-*mrpH* as the template. Then, the template DNA was digested with DpnI, and *E*. *coli* (strain NEB5α or NEB10β, New England Biolabs) was transformed with digested DNA. Successful mutagenesis was confirmed by DNA sequencing of plasmids from ampicillin-resistant clones, and *P*. *mirabilis mrpI*-ON *mrpH* was electroporated with the mutated plasmids. Double or triple mutations were constructed by subsequent rounds of mutagenesis on single or double mutated plasmids, respectively. Ampicillin (100 μg/ml) was used to maintain plasmids in biofilm assays with complemented strains of bacteria.

### Immunoblots

Bacteria were cultured statically at 37°C in 5 ml of LB for 48 h, then adjusted to OD_600_ = 0.8. One ml of culture was collected by centrifugation. Acid treatment was used to dissociate fimbrial subunits [99,100] prior to SDS-PAGE and immunoblot. Briefly, pellets were resuspended in 80 μl of distilled water, pH 1.8, and incubated at 95°C for 10 min. 6X SDS-PAGE sample buffer was added, and the pH was then neutralized by addition of 0.5 μl increments of 1N NaOH. A 15% polyacrylamide gel was loaded with 10 μl per lane of sample. Proteins were separated by electrophoresis, transferred to PVDF membrane, and probed for MrpH with affinity-purified anti-MrpH polyclonal antibody [23]. After chemiluminescent detection, blots were stripped and reprobed with anti-MrpA polyclonal antibody [23]. A replicate polyacrylamide gel was stained with Coomassie blue to assess protein loading.

### Hemagglutination assays

To examine the ability of *P*. *mirabilis* to hemagglutinate red blood cells, a modification of Li’s protocol was used [23,99]. Guinea pig erythrocytes in Alsever’s solution were collected by centrifugation at 2800 x *g* for 5 min and washed three times with ice-cold PBS before being suspended in PBS at a final concentration of 3%. Bacteria were cultured statically at 37°C in 5 ml of LB for 48 h, then adjusted to OD_600_ = 0.8. Two ml of culture was collected by centrifugation, and bacteria were resuspended in 100 μl of PBS. Serial two-fold dilutions of bacteria were prepared in PBS in a 96-well polyvinyl chloride U-bottom well plate, with 25 μl of bacterial suspension per well. Negative control wells included only PBS. Then, an equal volume of 3% erythrocytes was added to each well and mixed well. After allowing 30 min to settle, hemagglutination was recorded.

### Ethics statement

Animal experiments were approved by the University of Michigan Medical School Institutional Animal Care and Use Committee, protocol number PRO00007111. During catheterization procedures, mice were anesthetized by intraperitoneal injection of ketamine/xylazine. Mice were euthanized by inhalant isoflurane anesthetic overdose prior to organ removal.

### Mouse model of ascending UTI

To assess the fitness of the *mrpH* H72A mutation, an adaptation [23] of a mouse model of ascending UTI [101] was used [102]. Briefly, a kanamycin-sensitive *mrpH* mutant carrying pGEN-MCS (empty vector [98]) and an isogenic kanamycin-resistant *mrpH* mutant complemented with either wild-type *mrpH* or *mrpH* H72A were cultured overnight and individually adjusted to an estimated density of 2 x 10^8^ CFU/ml (OD_600_ = 0.2). Cultures of each complemented strain were mixed in a 1:1 ratio with the empty vector strain. Using a Harvard Apparatus infusion pump, female CBA/J mice (age 5–6 weeks) were transurethrally inoculated with a 50 μl suspension containing 1 x 10^7^ CFU of this 1:1 mixture of bacteria. At 7 days postinoculation, urine was collected, and bladders, kidneys, and spleens were harvested. Organs were homogenized (OMNI International) and spiral plated (Autoplate 4000; Spiral Biotech) onto both LB agar and LB agar supplemented with kanamycin to enumerate wild-type and mutant CFU (Qcount, Spiral Biotech). Selected dilutions were also plated on LB agar supplemented with ampicillin to confirm plasmid retention. The competitive index (CI) was calculated by dividing the ratio of complemented strain to empty vector output CFU to the ratio of complemented to empty vector input CFU; log CI > 0 indicates increased fitness of the complemented strain compared to the empty vector control.

### Statistics and software

Biofilm, hemagglutination, and mouse model data were plotted and statistics calculated using GraphPad Prism 7. For all biofilm and hemagglutination data, error bars show SD. To calculate statistical significance for these assays, one-way ANOVA with Dunnett’s multiple comparisons test was used. For mouse cochallenge experiments, statistical significance was calculated using the two-tailed Wilcoxon matched-pairs test. *P < 0.05, **P < 0.01, ***P < 0.001, ****P < 0.0001.

## Supporting information

S1 Table Constructs and Protein Science Facility screening results. (DOCX)

S2 Table Strains and plasmids used in this study. (DOCX)

S3 Table SAD data collection and phasing statistics. (DOCX)

S4 Table Primers used in this study. (DOCX)

S1 Fig Comparison between the 3D structures of MrpH_153_ and MrpH_159_. Structures are shown as cartoon with MrpH_153_ in blue and MrpH_159_ in yellow. Three prolines in the C-terminus of MrpH_159_, and the His-tag in MrpH_153_, are shown as stick, with carbon atoms in the same color as the cartoon. Three conserved histidine residues and two disulfide bonds are also shown as stick. Zn^2+^ ions are shown as spheres in purple for MrpH_153_ and in green for MrpH_159_.
(TIF)

S2 Fig Difference electron density next to the Zn^2+^ in MrpH_153_ and MrpH_159_. Difference electron density next to the Zn^2+^ in MrpH_153_ (A) and MrpH_159_ (B). (A) The three metal-coordinating histidine side chains and a molecule of tartrate are shown as labelled stick models together with m*Fo*-D*Fc* electron density calculated before modelling in tartrate. (B) Side chains of the three metal-coordinating histidine residues and of Arg 118 (in two alternative conformations), and a molecule of glutamic acid are shown as labelled stick models together with m*Fo*-D*Fc* electron density calculated before modelling in glutamic acid. In both (A) and (B), m*Fo*-D*Fc* electron density contoured at 3.0 σ above the mean is shown as a green mesh.
(TIF)

S3 Fig TXRF spectra. TXRF spectra of (A) MrpH_159_ and (B) buffer control show that MrpH_ntd_ binds zinc.
(TIF)

S4 Fig MrpH_ntd_ thermal stability is increased at acidic pH. (TIF)

S5 Fig Tartrate, glutamate, and ionic strength have no effect on biofilm formation. (A) Although tartrate (Tar) co-crystallized with MrpH_153_ and glutamate (Glu) is consistent with MrpH_159_, addition of either substrate to biofilm cultures as a potential competitive inhibitor resulted in no change. Addition of other similar substrates (aspartate, glutamine, or asparagine) also had no effect on biofilm formation. (B) Addition of NaCl as a competitor of electrostatic interactions had no effect on biofilm formation. Note that NaCl experiments used *mrpI*-ON instead of L-ON as the positive control.
(TIF)

S6 Fig EDTA and TPEN inhibit MR/P-dependent biofilm formation. (A and B) Growth curves of L-ON or L-OFF in Minimal A pH 6 show the disruption of biofilm formation by L-ON in the presence of 1 μM EDTA (A) or TPEN (B), resulting in a classic, smooth planktonic curve. Ethanol (EtOH), used as a vehicle for TPEN, had no effect on L-ON growth kinetics. (C and D) Growth curves of L-ON in Minimal A pH 6 with increasing concentrations of EDTA (C) or TPEN (D) showing chelator levels that impede growth. L-OFF with no chelator is included as a control. Note that the curves in D are from the same experiment as the curves in B. (E) Crystal violet biofilm assays of *P*. *mirabilis* L-OFF cultured in the presence of metal chelators. Addition of chelators had no effect on background biofilm formation by L-OFF. DF, deferoxamine; ns, not significant. (F) Biofilm assays of wild-type *P*. *mirabilis* HI4320 cultured in Minimal A pH 6 with or without 50 μM ZnSO_4_ added.
(TIF)

S7 Fig Additional chelation biofilm experiments. (A) Identification of metal-restricted culture conditions for metal complementation experiments. Wild-type *P*. *mirabilis* primarily grows planktonically under these conditions, and acts as a control for overall growth. Culture conditions were as follows. LB to Min A: overnight culture in LB followed by 22 h culture in untreated Minimal A pH 6; this is the standard condition used for most biofilm assays in this study. Chelexed MinA: overnight culture in LB, followed by two washes in Minimal A and subsequent 22 h culture in chelexed Minimal A, pH 6. Chelexed + 0.0002% CAA: same, but medium was supplemented with 0.0002% chelexed casamino acids. (B) Biofilm formation by L-ON in unchelexed Minimal A. Addition of 50 μM ZnSO_4_ overcomes biofilm inhibition due to 1 μM TPEN. *P < 0.05 vs. untreated by one-way ANOVA with Dunnett’s multiple comparisons test.
(TIF)

S8 Fig Mutation of multiple His residues has no further effect compared with single mutations. Compare with Fig 11. Biofilm formation by an MR/P locked-ON mutant, a locked-ON *mrpH* double mutant, and the double mutant complemented with wild-type or mutated *mrpH* expressed from plasmid pGEN-Pmrp-*mrpH* (+*mrpH*). Mutating any of the Zn-coordinating histidine residues (His 72, His 74, His 117) to alanine, alone or in combination, completely abolished *P*. *mirabilis* biofilm formation. pGEN-Pmrp-*luxCDABE* (EV) is a negative control plasmid and has luciferase genes under the control of the native *mrp* promoter. All columns, including *mrpI*-ON positive control, are *P* < 0.0001 *vs*. pGEN-Pmrp-*mrpH* by one-way ANOVA with Dunnett’s multiple comparisons test.
(TIF)

S9 Fig Immunoblots of MrpH and MrpA. (A) Whole cell lysates of 48h static cultures were subjected to acid treatment to dissociate fimbrial subunits and separated by SDS-PAGE. Following detection of MrpH, blots were stripped and reprobed with anti-MrpA antibodies. Lanes 1–6 are *P*. *mirabilis*; lane 1 is *mrpI-*ON, and lanes 2–6 are *mrpI-*ON Δ*mrpH*. Lane 2, no plasmid; lane 3, pGEN-Pmrp-*mrpH*; lanes 4–6, site-directed mutants as indicated. Lanes 7–8 are *E*. *coli* BW25113Δ*fimA* containing either plasmid pXL4401 (*mrpA-G*) or pXL1305 (*mrpA-H*). Molecular size markers in kilodaltons are shown on the left. (B) a replicate polyacrylamide gel was stained with Coomassie blue to assess protein loading.
(TIF)

S10 Fig Residues selected for site-directed alanine substitutions. MrpH_ntd_ is shown as a ribbon with targeted residues shown as stick models and labeled. (A) side view, (B) top view.
(TIF)

S11 Fig Production of MR/P fimbriae in afimbriate *E*. *coli* did not lead to biofilm formation. (A) Biofilm formation by *P*. *mirabilis* HI4320 mutants (left) and *E*. *coli* BW25113Δ*fimA* with plasmids pXL1305 (*mrpA-H*) or pXL4401 (*mrpA-G*) (right). (B) HA by the same *P*. *mirabilis* and *E*. *coli* strains (representative experiments). (C and D) Quantification of *E*. *coli* biofilm formation and HA, respectively (n = 6 independent replicates). Panel D shows the same data as in Fig 12B and is included here to facilitate comparison.
(TIF)
