# Supplementary material for: MrpH, a new class of metal-binding adhesin, requires zinc to mediate biofilm formation
Source: PLoS Pathog. 2020 Aug 11;16(8):e1008707. doi: 10.1371/journal.ppat.1008707 (PMC7444556; doi:10.1371/journal.ppat.1008707)
Supplement: S1 Table — (DOCX) [file ppat.1008707.s001.docx]

**S1 Table** Constructs and Protein Science Facility screening results

| *Construct* | *Start residue* | *End residue* | *Soluble*  *small-scale expression^*^* | *Comments* |
| --- | --- | --- | --- | --- |
| wuMrpHm | M1 | V159 | - | Purified MrpH_159_ protein: MrpH(25-159)-HHHHHH |
| psfMRPH-c001 | M1 | G153 | 3 | Purified MrpH_153_ protein: MrpH(25-153)-AHHHHHH |
| psfMRPH-c002 | M1 | P156 | 3 |  |
| psfMRPH-c003 | M1 | G160 | 3 |  |

* Amounts of both totally expressed and purified protein was analyzed on SDS-PAGE. Based on the SDS-PAGE results, the soluble expression levels were scored as 4: dominating band of target protein; 3: band much stronger than background; 2: band equal to / stronger than background; 1: band weaker than background; 0: no detected protein.
