# Supplementary material for: MrpH, a new class of metal-binding adhesin, requires zinc to mediate biofilm formation
Source: PLoS Pathog. 2020 Aug 11;16(8):e1008707. doi: 10.1371/journal.ppat.1008707 (PMC7444556; doi:10.1371/journal.ppat.1008707)
Supplement: S2 Table — (DOCX) [file ppat.1008707.s002.docx]

| **Supplementary Table S2. Strains and plasmids used in this study** | | |  |
| --- | --- | --- | --- |
|  |  |  |  |
| **Strains** | **Description** | **Source *^a^*** |  |
| ***E. coli*** |  |  |  |
| NEB5α | Host for routine cloning | New England Biolabs |  |
| NEB10β | Host for routine cloning | New England Biolabs |  |
| Top 10 | Host for routine cloning | Invitrogen |  |
| BW25113Δ*fimA* | Afimbriate *E. coli* from Keio collection | [1] |  |
| BL21-AI | Expression host | Invitrogen |  |
| ***P. mirabilis*** |  |  |  |
| HI4320 | Clinical isolate of *P. mirabilis* | [2] |  |
| HI4320 L-ON | Kanamycin-resistant *mrpI* mutant with *mrp* promoter invertible element locked in the "ON" orientation | [3] |  |
| HI4320 L-OFF | Kanamycin-resistant *mrpI* mutant with *mrp* promoter invertible element locked in the "OFF" orientation | [3] |  |
| HI4320 *mrpI*-ON | Kanamycin-resistant *mrpI* mutant with *mrp* promoter invertible element locked in the "ON" orientation; constructed using targetron method | This study |  |
| HI4320 *mrpI*-ON Δ*mrpH* | Kanamycin-resistant *mrpI mrpH* double mutant | This study |  |
| HI4320 *mrpH*Ωkan | Kanamycin-resistant *mrpH* mutant | This study |  |
| HI4320 *mrpH*Δkan | Kanamycin-sensitive *mrpH* mutant | This study |  |
|  |  |  |  |
| **Plasmids** |  |  |  |
| **Name** | **Description** | **Antibiotic(s)** | **Source *^a^*** |
| wuMrpHm | Encodes HI4320 MrpH residues 1-159 + C-terminal His_6_-tag | Kan^R^ | This study |
| psfMRPH-c001 | Encodes HI4320 MrpH residues 1-153 + A + C-terminal His6-tag | Amp^R^ | This study |
| pAR1219 | Encodes inducible T7 polymerase | Amp^R^ | Sigma-Aldrich |
| pQL123 | Encodes *cre*-recombinase | Amp^R^ | [4] |
| pACD4K-CloxP | TargeTron Gene Knockout System plasmid; contains retargetable intron for constructing mutations | Chl^R^ | Sigma-Aldrich |
| pANN126 | Targetron plasmid for mutating *mrpH*; backbone is pACD4K-CloxP | Chl^R^ | This study |
| pANN128 | Targetron plasmid for mutating *mrpI*; backbone is pACD4K-CloxP | Chl^R^ | This study |
| pGEN-MCS | Low copy number cloning vector; p15A ori; *par hok sok mok parM parR* | Amp^R^ | [5] |
| pGEN-Pmrp-*luxCDABE* | pJS027. The *mrp* promoter from *P. mirabilis* (from the end of *mrpI* to the ATG) in front of *lux* in pGEN cloned using KpnI and BamHI sites | Amp^R^ | This study |
| pGEN-Pmrp-*mrpH* | pJS085. *mrpH* cloned into the BamHI /SalI site of pGEN-mrp-lux (pJS027) to remove *lux* | Amp^R^ | This study |
| pMB-H72A | pJS085 with H72A mutation | Amp^R^ | This study |
| pMB-H74A | pJS085 with H74A mutation | Amp^R^ | This study |
| pMB-H117A | pJS085 with H117A mutation | Amp^R^ | This study |
| pMB-H72.74A | pJS085 with H72A and H74A mutations, constructed from pMB-H72A | Amp^R^ | This study |
| pMB-H72.117A | pJS085 with H72A and H117A mutations, constructed from pMB-H72A | Amp^R^ | This study |
| pMB-H74.117A | pJS085 with H74A and H117A mutations, constructed from pMB-H74A | Amp^R^ | This study |
| pMB-H72.74.117A | pJS085 with H72A, H74A, H117A mutations; constructed from pMB-H72.74A | Amp^R^ | This study |
| pMB-N82A | pJS085 with N82A mutation | Amp^R^ | This study |
| pMB-K92A | pJS085 with K92A mutation | Amp^R^ | This study |
| pMB-R94A | pJS085 with R94A mutation | Amp^R^ | This study |
| pMB-T116A | pJS085 with T116A mutation | Amp^R^ | This study |
| pMB-R118A | pJS085 with R118A mutation | Amp^R^ | This study |
| pMB-E127A | pJS085 with E127A mutation | Amp^R^ | This study |
| pMB-C128A | pJS085 with C128A mutation | Amp^R^ | This study |
| pMB-R143A | pJS085 with R143A mutation | Amp^R^ | This study |
| pMB-K145A | pJS085 with K145A mutation | Amp^R^ | This study |
| pMB-R143.K145A | pJS085 with R143A and K145A mutations; constructed using pMB-R143A as the template | Amp^R^ | This study |
| pXL1305 | pBluescript-L-ON *mrpA-H* (wt was mutated to C128S/C152S then re-mutated back to WT) | Amp^R^ | [6] |
| pXL4401 | pBluescript-L-ON-*mrpA-G* (with *mrpH* deleted) | Amp^R^ | [6] |
| pXL1305-H72A | *mrpA-H* with H72A mutation in *mrpH* | Amp^R^ | This study |
| *^a^* **References** |  |  |  |
| 1. Baba T, Ara T, Hasegawa M, Takai Y, Okumura Y, Baba M, et al. Construction of *Escherichia coli* K-12 in-frame, single-gene knockout mutants: the Keio collection. Mol Syst Biol. 2006;2:2006 0008. Epub 2006/06/02. doi: 10.1038/msb4100050. PubMed PMID: 16738554; PubMed Central PMCID: PMC1681482 | | | |
| 2. Warren JW, Tenney JH, Hoopes JM, Muncie HL, Anthony WC. A prospective microbiologic study of bacteriuria in patients with chronic indwelling urethral catheters. Journal of Infectious Diseases. 1982;146(6):719-23. PubMed PMID: 6136. | | | |
| 3. Li X, Lockatell CV, Johnson DE, Mobley HLT. Identification of MrpI as the sole recombinase that regulates the phase variation of MR/P fimbria, a bladder colonization factor of uropathogenic *Proteus mirabilis*. Molecular Microbiology. 2002;45(3):865-74. PubMed PMID: 5707. | | | |
| 4. Enyeart PJ, Chirieleison SM, Dao MN, Perutka J, Quandt EM, Yao J, et al. Generalized bacterial genome editing using mobile group II introns and Cre-lox. Mol Syst Biol. 2013;9:685. Epub 2013/09/05. doi: 10.1038/msb.2013.41. PubMed PMID: 24002656; PubMed Central PMCID: PMC3792343. | | | |
| 5. Lane MC, Alteri CJ, Smith SN, Mobley HLT. Expression of flagella is coincident with uropathogenic *Escherichia coli* ascension to the upper urinary tract. Proc Natl Acad Sci U S A. 2007;104(42):16669-74. PubMed PMID: 17925449. | | | |
| 6. Li X, Johnson DE, Mobley HLT. Requirement of MrpH for mannose-resistant *Proteus*-like fimbria-mediated hemagglutination by *Proteus mirabilis*. Infection and Immunity. 1999;67(6):2822-33. PubMed PMID: 5541 | | | |
