# Supplementary material for: MrpH, a new class of metal-binding adhesin, requires zinc to mediate biofilm formation
Source: PLoS Pathog. 2020 Aug 11;16(8):e1008707. doi: 10.1371/journal.ppat.1008707 (PMC7444556; doi:10.1371/journal.ppat.1008707)
Supplement: S3 Table — (DOCX) [file ppat.1008707.s003.docx]

**Table S3.** SAD data collection and phasing statistics

|  | **MrpH_153_ Pt derivative** |
| --- | --- |
| Data collection |  |
| Space group | P2_1_ |
| *a, b, c* (Å) | 25.58, 53.28, 40.39 |
| *α, β, γ* (°) | 90.0, 103.43, 90.0 |
| Wavelength (Å) | 1.072 |
| Resolution (Å) | 39.29-1.26 (1.305-1.26) |
| Total reflections | 185285 (17677) |
| Unique reflections | 52844 (5042) |
| Anomalous multiplicity | 3.5 (3.5) |
| Anomalous completeness | 94.21 (90.08) |
| *<I/σ(I)>* | 12.62 (1.87) |
| Wilson B-factor (Å^2^) | 12.04 |
| *R_merge_ (within I+/I-)* | 0.051 (0.612) |
| *R_meas_ (within I+/I-)* | 0.060 (0.721) |
| *R_pim_ (within I+/I-)* | 0.031 (0.378) |
| *CC_1/2_* | 0.999 (0.832) |
| *CC_1/2_(Δ_anom_)* | 0.310 (0.140) |
| Anomalous signal *(ΔF_anom_/σ(ΔF_anom_))* | 1.135 (0.873) |
| Phasing |  |
| No of sites | 2 |
| <Figure-of-merit> | 0.329 |
