# Supplementary material for: MrpH, a new class of metal-binding adhesin, requires zinc to mediate biofilm formation
Source: PLoS Pathog. 2020 Aug 11;16(8):e1008707. doi: 10.1371/journal.ppat.1008707 (PMC7444556; doi:10.1371/journal.ppat.1008707)
Supplement: S4 Table — (DOCX) [file ppat.1008707.s004.docx]

| **Supplementary Table S4. Primers used in this study** | |  |
| --- | --- | --- |
| **Name** | **Description** | **Sequence** |
| pGEN-5' | Used to sequence pGEN-Pmrp-*mrpH* | GAATTCCTGCAGGGCATG |
| Pmrp-for | Used to sequence pGEN-Pmrp-*mrpH* | TTCGCGGCGTTTGAAGTG |
| pGEN-3'#2 | Used to sequence pGEN-Pmrp-*mrpH* | GTAGAGCTCATCCATGCC |
| mrpH3'-out | Used to sequence pGEN-Pmrp-*mrpH* | GTGAGTTCAACGCTACGCAC |
| Pm IE P1 | Used with PCR to assess the orientation of the *mrp* promoter invertible element | GCATCAATAAAGGGTTGTGTTTT |
| Pm IE P2 | Used with PCR to assess the orientation of the *mrp* promoter invertible element | ATTGATGCTCCTTGCTCAATTAC |
| IE ON | Used to assess if invertible element is ON | TTCTCCGGCTTCTAAGTC |
| IE OFF | Used to assess if invertible element is OFF | GTAATTGAGCAAGGAGCATCAAT |
| mrpI-TT-F | Used to PCR confirm targetron insertions in *mrpI* | CATGGTTTTCGTGTTAGCG |
| mrpI-TT-R | Used to PCR confirm targetron insertions in *mrpI* | GTGTTGTTGAAAGCCCACC |
| mrpH Seq F | To check if kan casette was excised from *mrpH* | TTCTCAGCACCTGCGATGGC |
| mrpH Seq R | To check if kan casette was excised from *mrpH* | GGAACCGGAGAAACGACCTGG |
| mrpJ-RTR | Sequencing primer reading into *mrpH* | TGTTCATACCTCGTTCATAGCG |
| mrpH-IBS | For mutating targetron to target *mrpH* | AAAAAAGCTTATAATTATCCTTAGGGGGCAATCCTGTGCGCCCAGATAGGGTG |
| mrpH-EBS1d | For mutating targetron to target *mrpH* | CAGATTGTACAAATGTGGTGATAACAGATAAGTCAATCCTGCTAACTTACCTTTCTTTGT |
| mrpH-EBS2 | For mutating targetron to target *mrpH* | TGAACGCAAGTTTCTAATTTCGATTCCCCCTCGATAGAGGAAAGTGTCT |
| mrpI-IBS | For mutating targetron to target *mrpI* | AAAAAAGCTTATAATTATCCTTAAGTAGCATACTCGTGCGCCCAGATAGGGTG |
| mrpI-EBS1d | For mutating targetron to target *mrpI* | CAGATTGTACAAATGTGGTGATAACAGATAAGTCATACTCTAAAACTTACCTTTCTTTGT |
| mrpI-EBS2 | For mutating targetron to target *mrpI* | TGAACGCAAGTTTCTAATTTCGATTCTACTTCGATAGAGGAAAGTGTCT |
| EBS Universal | Univeral primer for targetron mutagenesis | AATTAGAAACTTGCGTTCAGTAAACACAACTTATAC |
| M13f | For sequencing inserts in pCR2.1 vector | GTAAAACGACGGCCAG |
| M13r | For sequencing inserts in pCR2.1 vector | CAGGAAACAGCTATGAC |
| pACD4K 5' | Confirmation of intron cloning in pACD4K-CloxP | CCGCGAAATTAATACGACTCACTA |
| pACD4K 3' | Confirmation of intron cloning in pACD4K-CloxP | GGTATCCCCAGTTAGTGTTA |
|  |  |  |
| **Site-directed mutagenesis primers *^a^*** | |  |
| H72A QuikF | Construction of H72A mutant | GTGTGTTATGTCACAGTGAAC**GCT**AAACATACAGTAAATGGTAC |
| H72A QuikR | Construction of H72A mutant | GTACCATTTACTGTATGTTT**AGC**GTTCACTGTGACATAACACAC |
| H74A QuikF | Construction of H74A mutant | TGTCACAGTGAACCATAAA**GCT**ACAGTAAATGGTACTGGG |
| H74A QuikR | Construction of H74A mutant | CCCAGTACCATTTACTGT**AGC**TTTATGGTTCACTGTGACA |
| H117A QuikF2 | Construction of H117A mutant | CAAACCAC**CGC**CAGAGGGCCTTCATTAAACTC |
| H117A QuikR2 | Construction of H117A mutant | GCCCTCTG**GCG**GTGGTTTGCCCTTCAATAG |
| H72A H74A QuikF | Construction of H72A H74A double mutant, using H72A as template | TGTCACAGTGAACGCTAAA**GCT**ACAGTAAATGGTACTGGG |
| H72A H74A QuikR | Construction of H72A H74A double mutant, using H72A as template | CCCAGTACCATTTACTGT**AGC**TTTAGCGTTCACTGTGAC |
| N82A for | Construction of N82A mutant | AGTAAATGGTACTGGGGGA**GCT**CCTGCATTTCAGATTGCT |
| N82A rev | Construction of N82A mutant | AGCAATCTGAAATGCAGG**AGC**TCCCCCAGTACCATTTACT |
| K92A for3 | Construction of K92A mutant | CCTGCATTTCAGATTGCTCGAATTGAA**GCA**CTACGTACTT |
| K92A rev3 | Construction of K92A mutant | CACGAACTTCAGCTAAAGTACGTAG**TGC**TTCAATTCGAGC |
| R94A for | Construction of R94A mutant | GCTCGAATTGAAAAACTA**GCT**ACTTTAGCTGAAGTTCGTG |
| R94A rev | Construction of R94A mutant | CACGAACTTCAGCTAAAGT**AGC**TAGTTTTTCAATTCGAGC |
| T116A for2 | Construction of T116A mutant | GCAAACC**GCC**CACAGAGGGCCTTCATTAAACTC |
| T116A rev2 | Construction of T116A mutant | CCCTCTGTG**GGC**GGTTTGCCCTTCAATAGGGAA |
| R118A for3 | Construction of R118A mutant | CCCTATTGAAGGGCAAACCACCCAC**GCA**GGGCCTTCAT |
| R118A rev3 | Construction of R118A mutant | TTGATTAGAGTTTAATGAAGGCCC**TGC**GTGGGTGGTT |
| E127A for | Construction of E127A mutant | GGCCTTCATTAAACTCTAATCAA**GCG**TGTGTGGGATTATT |
| E127A rev | Construction of E127A mutant | AATAATCCCACACA**CGC**TTGATTAGAGTTTAATGAAGGCC |
| C128A for2 | Construction of C128A mutant | TAATCAAGAG**GCT**GTGGGATTATTTTATCAACCG |
| C128A rev2 | Construction of C128A mutant | ATAATCCCAC**AGC**CTCTTGATTAGAGTTTAATGA |
| R143Af | Construction of R143A mutant | TCAAGTGGTATATCACCT**GCA**GGAAAACTCTTACCAGGTTC |
| R143Ar | Construction of R143A mutant | GAACCTGGTAAGAGTTTTCC**TGC**AGGTGATATACCACTTGA |
| K145 for2 | Construction of K145A mutant | TTCAAGTGGTATATCACCTCGAGGA**GCA**CTCTTACCAG |
| K145 rev2 | Construction of K145A mutant | TACCGCATAGTGAACCTGGTAAGAG**TGC**TCCTCGAGGT |
| R143.K145 for | Construction of R143A K145A double mutant, using R143A as template | CAAGTGGTATATCACCTGCAGGA**GCA**CTCTTACCAGGTTC |
| R143.K145 rev | Construction of R143A K145A double mutant, using R143A as template | ATACCGCATAGTGAACCTGGTAAGAG**TGC**TCCTGCAGGTGA |

*^a^* Mutated residues are underlined; targeted codons are **bolded**.
