# Supplementary figures and images for: MrpH, a new class of metal-binding adhesin, requires zinc to mediate biofilm formation
Source: PLoS Pathog. 2020 Aug 11;16(8):e1008707. doi: 10.1371/journal.ppat.1008707 (PMC7444556; doi:10.1371/journal.ppat.1008707)

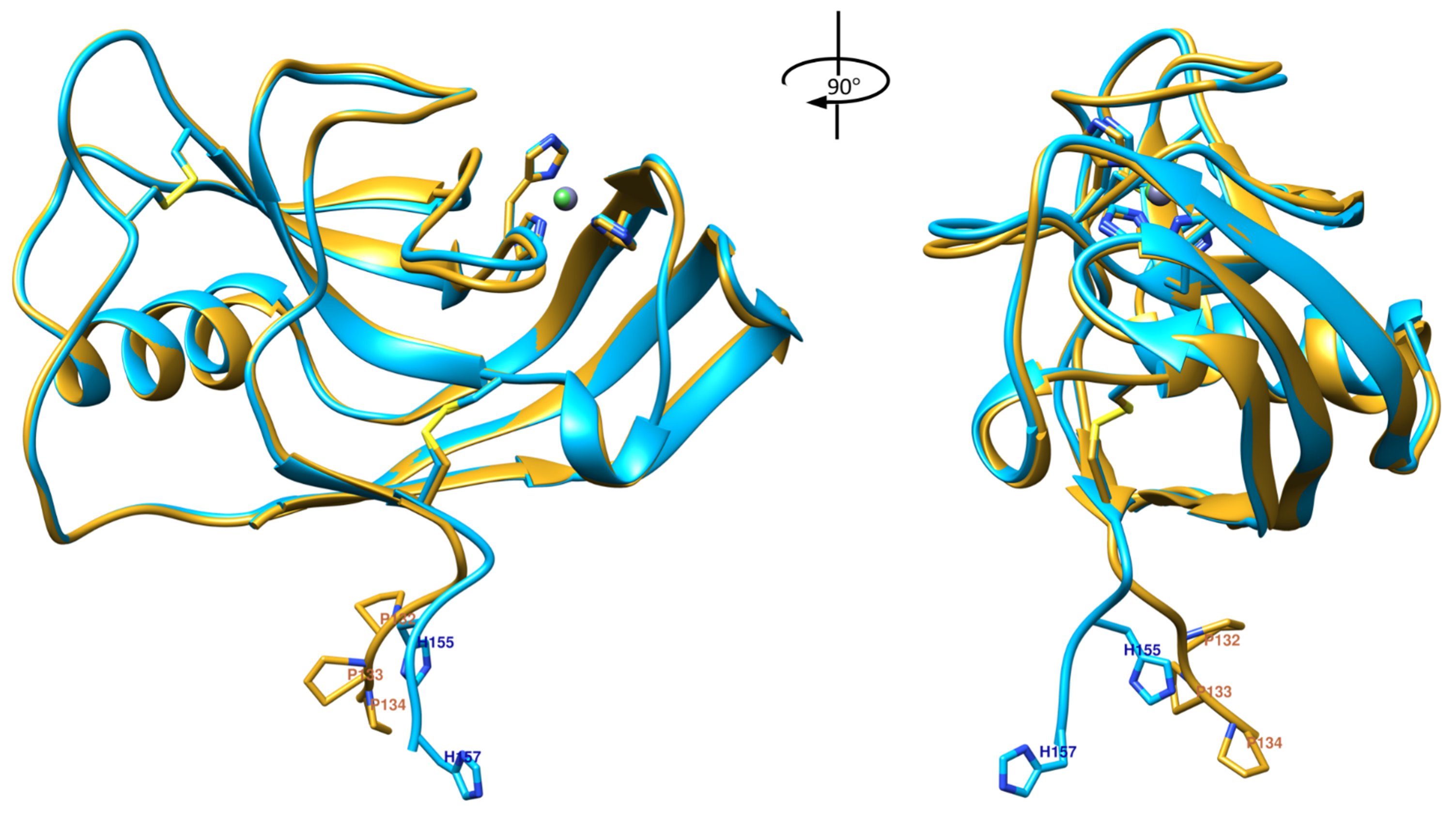

Supplement: S1 Fig — Structures are shown as cartoon with MrpH153 in blue and MrpH159 in yellow. Three prolines in the C-terminus of MrpH159, and the His-tag in MrpH153, are shown as stick, with carbon atoms in the same color as the cartoon. Three conserved histidine residues and two disulfide bonds are also shown as stick. Zn2+ ions are shown as spheres in purple for MrpH153 and in green for MrpH159. (TIF) [file ppat.1008707.s005.tif]

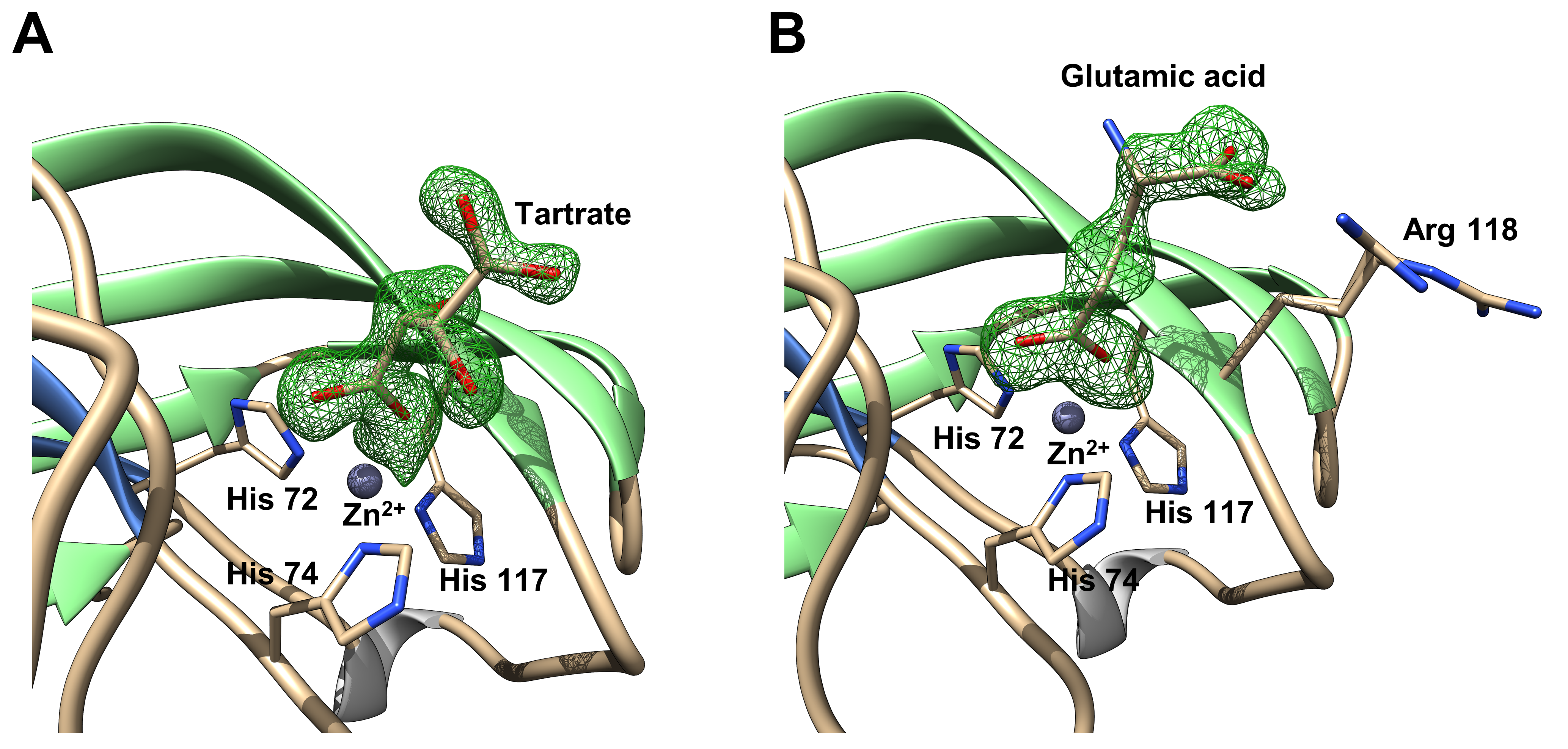

Supplement: S2 Fig — Difference electron density next to the Zn2+ in MrpH153 (A) and MrpH159 (B). (A) The three metal-coordinating histidine side chains and a molecule of tartrate are shown as labelled stick models together with mFo-DFc electron density calculated before modelling in tartrate. (B) Side chains of the three metal-coordinating histidine residues and of Arg 118 (in two alternative conformations), and a molecule of glutamic acid are shown as labelled stick models together with mFo-DFc electron density calculated before modelling in glutamic acid. In both (A) and (B), mFo-DFc electron density contoured at 3.0 σ above the mean is shown as a green mesh. (TIF) [file ppat.1008707.s006.tif]

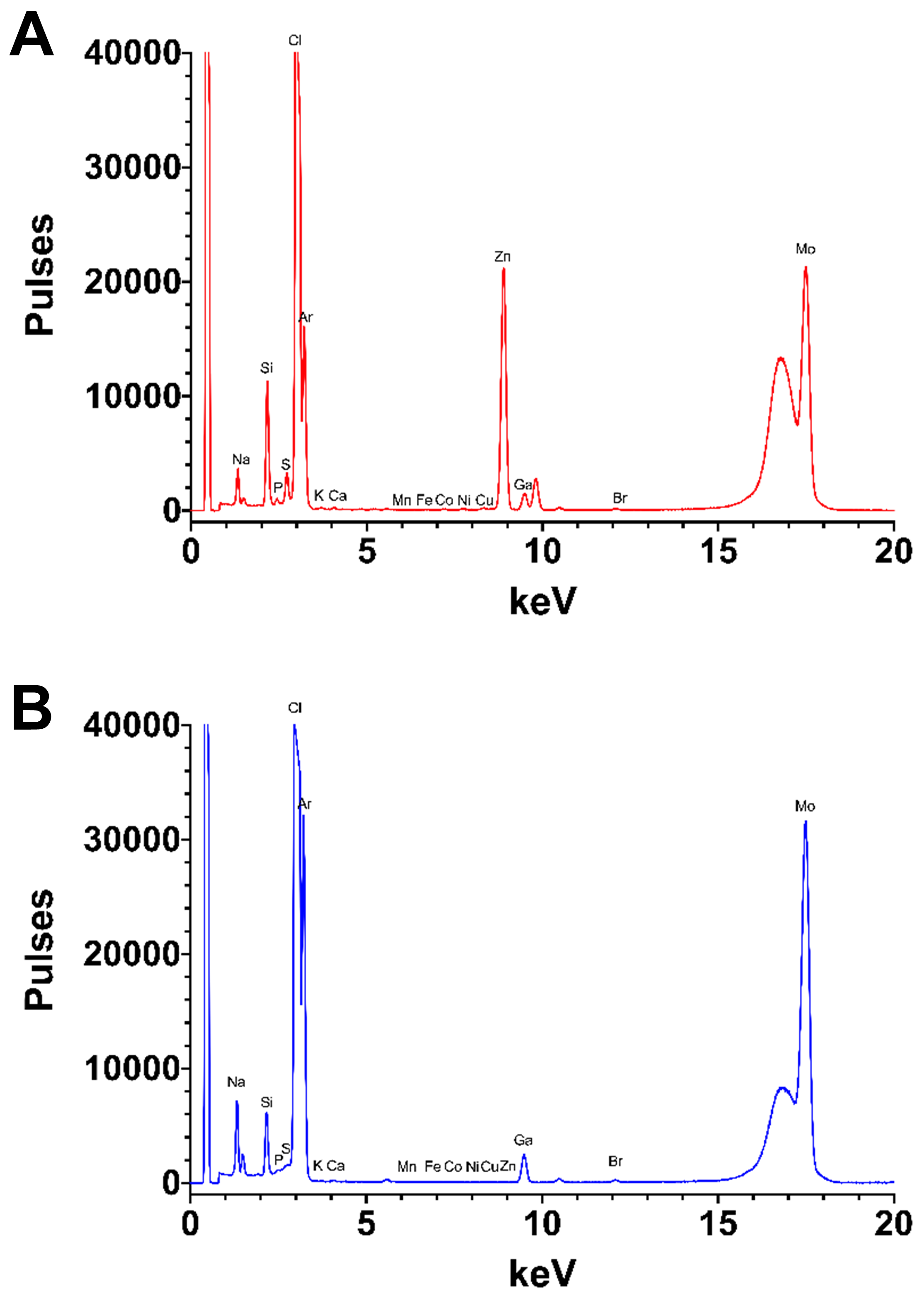

Supplement: S3 Fig — TXRF spectra of (A) MrpH159 and (B) buffer control show that MrpHntd binds zinc. (TIF) [file ppat.1008707.s007.tif]

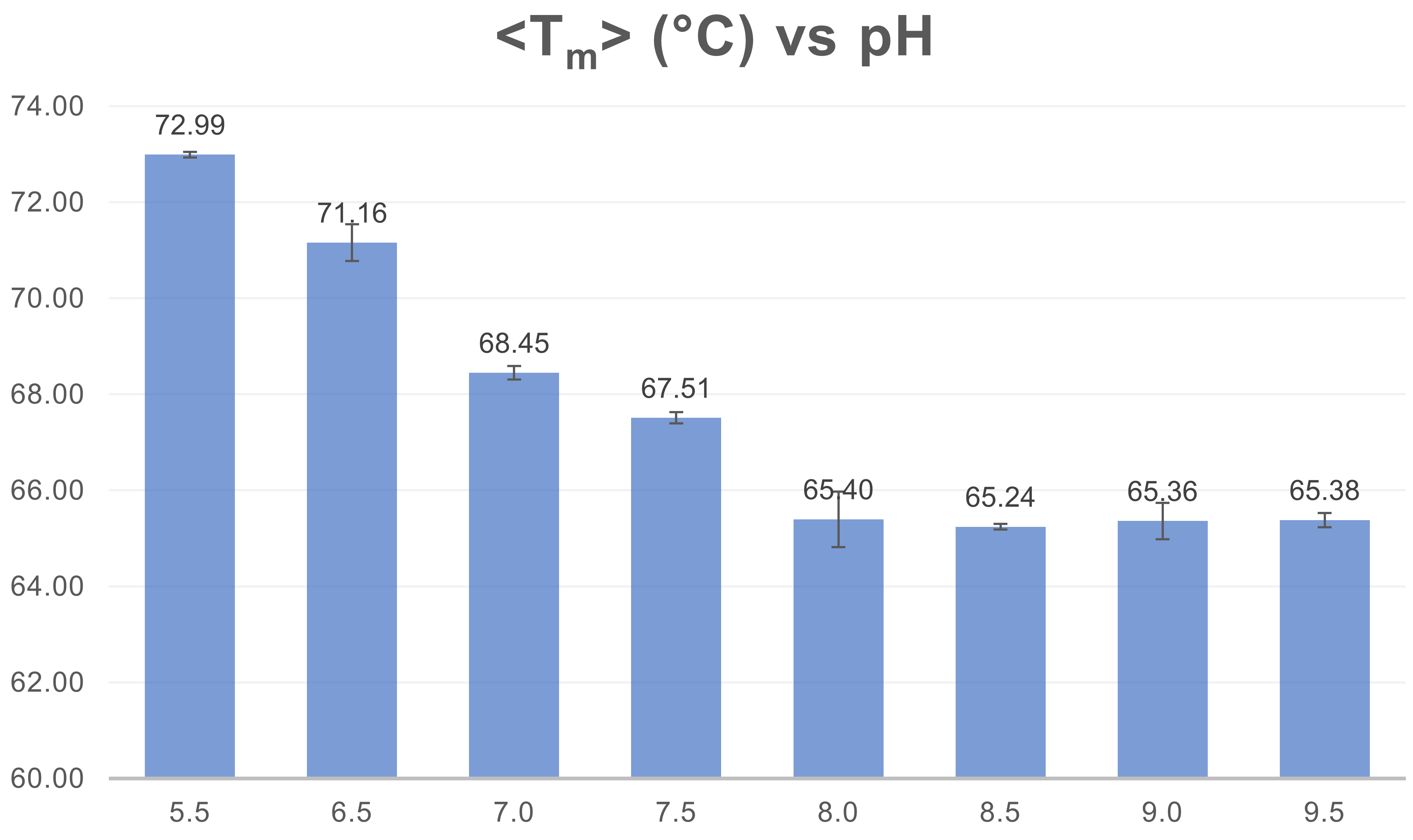

Supplement: S4 Fig — (TIF) [file ppat.1008707.s008.tif]

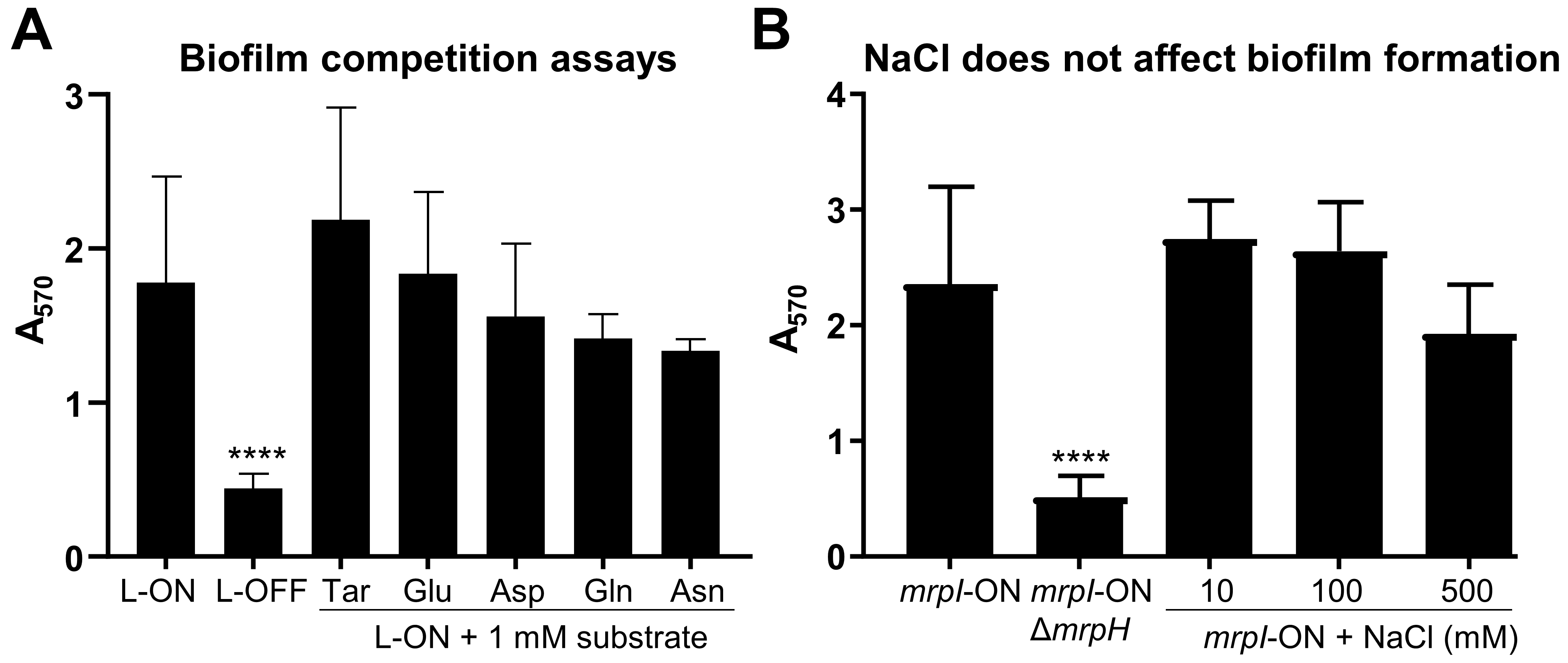

Supplement: S5 Fig — (A) Although tartrate (Tar) co-crystallized with MrpH153 and glutamate (Glu) is consistent with MrpH159, addition of either substrate to biofilm cultures as a potential competitive inhibitor resulted in no change. Addition of other similar substrates (aspartate, glutamine, or asparagine) also had no effect on biofilm formation. (B) Addition of NaCl as a competitor of electrostatic interactions had no effect on biofilm formation. Note that NaCl experiments used mrpI-ON instead of L-ON as the positive control. (TIF) [file ppat.1008707.s009.tif]

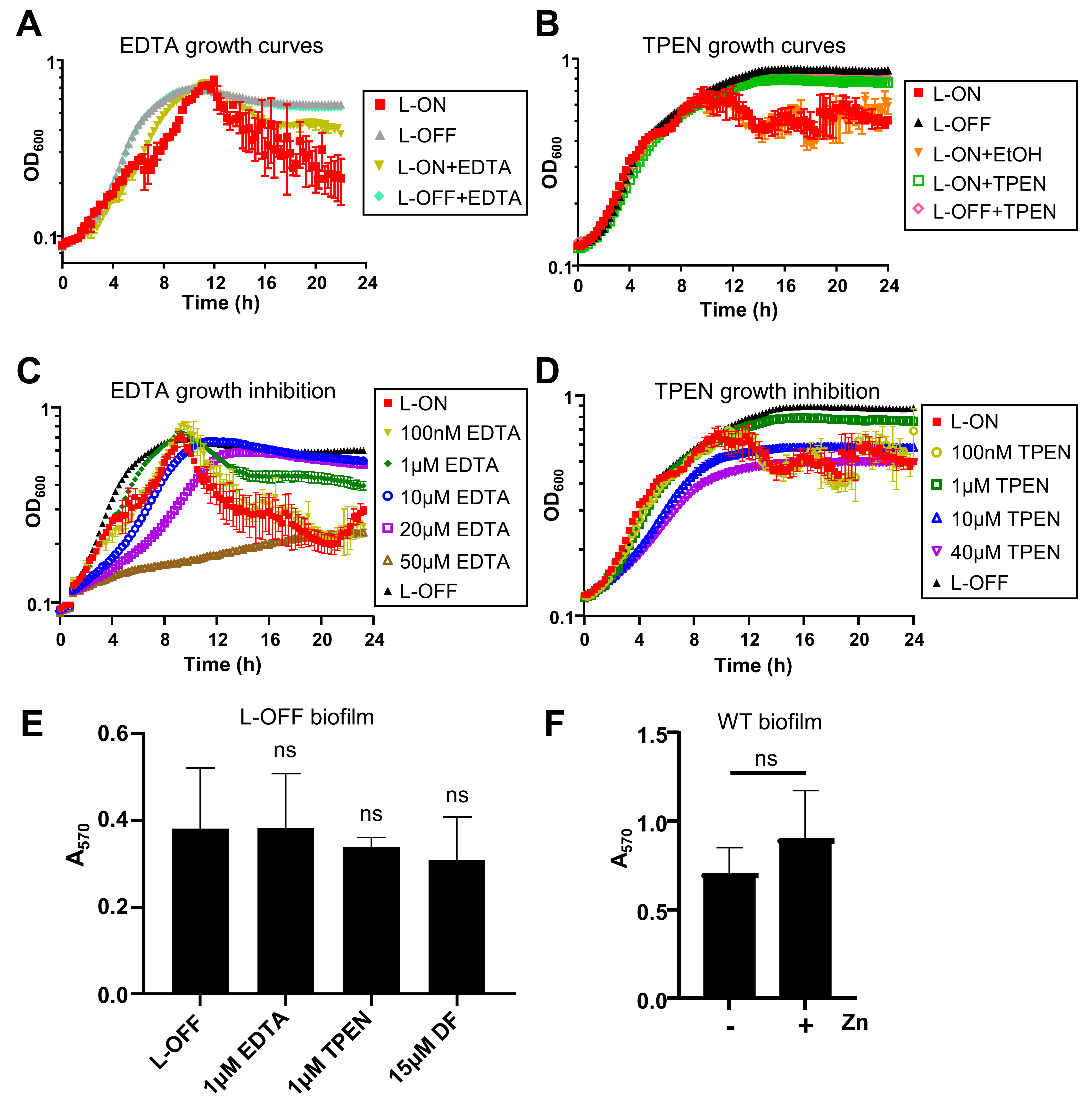

Supplement: S6 Fig — (A and B) Growth curves of L-ON or L-OFF in Minimal A pH 6 show the disruption of biofilm formation by L-ON in the presence of 1 μM EDTA (A) or TPEN (B), resulting in a classic, smooth planktonic curve. Ethanol (EtOH), used as a vehicle for TPEN, had no effect on L-ON growth kinetics. (C and D) Growth curves of L-ON in Minimal A pH 6 with increasing concentrations of EDTA (C) or TPEN (D) showing chelator levels that impede growth. L-OFF with no chelator is included as a control. Note that the curves in D are from the same experiment as the curves in B. (E) Crystal violet biofilm assays of P. mirabilis L-OFF cultured in the presence of metal chelators. Addition of chelators had no effect on background biofilm formation by L-OFF. DF, deferoxamine; ns, not significant. (F) Biofilm assays of wild-type P. mirabilis HI4320 cultured in Minimal A pH 6 with or without 50 μM ZnSO4 added. (TIF) [file ppat.1008707.s010.tif]

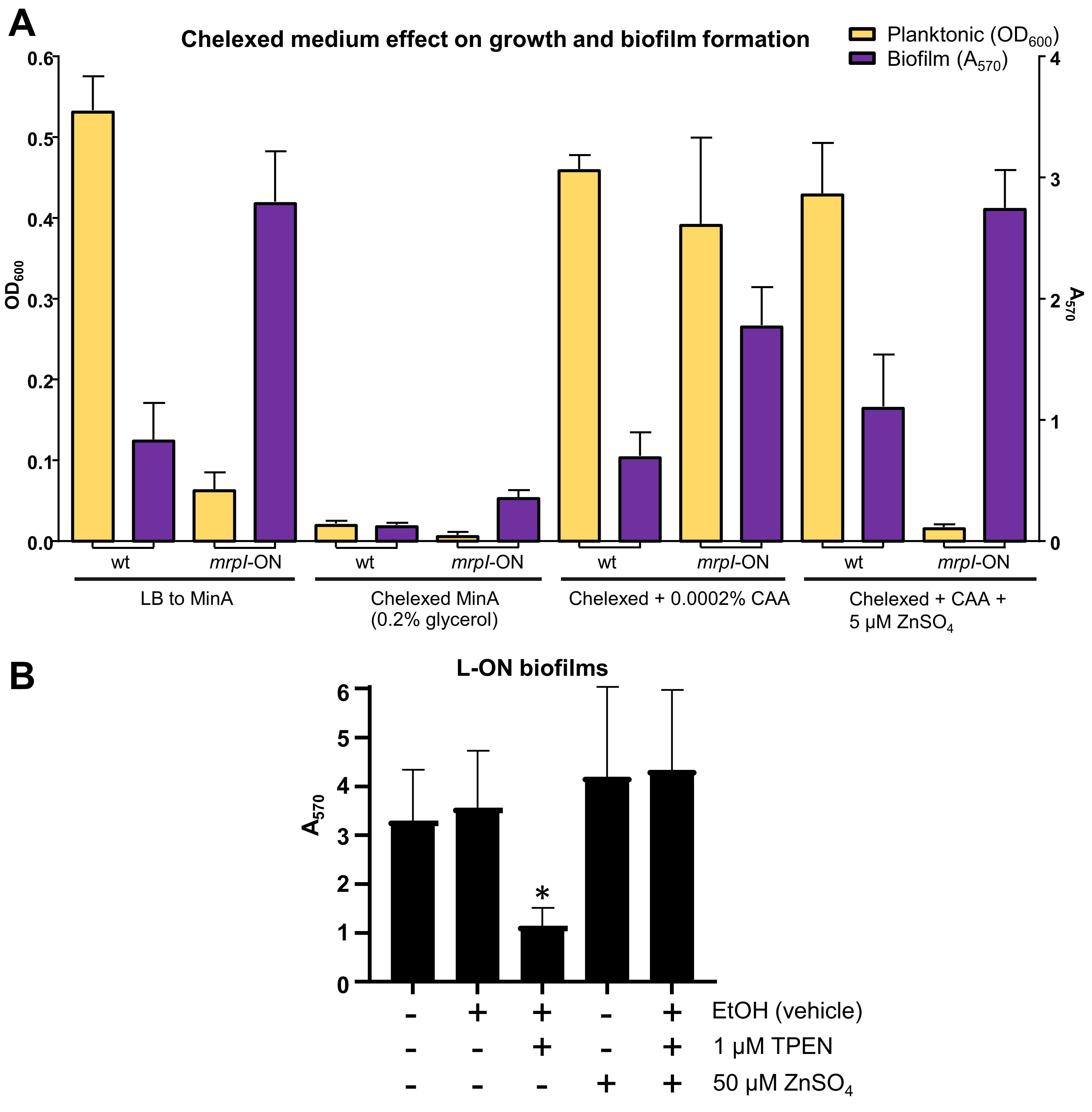

Supplement: S7 Fig — (A) Identification of metal-restricted culture conditions for metal complementation experiments. Wild-type P. mirabilis primarily grows planktonically under these conditions, and acts as a control for overall growth. Culture conditions were as follows. LB to Min A: overnight culture in LB followed by 22 h culture in untreated Minimal A pH 6; this is the standard condition used for most biofilm assays in this study. Chelexed MinA: overnight culture in LB, followed by two washes in Minimal A and subsequent 22 h culture in chelexed Minimal A, pH 6. Chelexed + 0.0002% CAA: same, but medium was supplemented with 0.0002% chelexed casamino acids. (B) Biofilm formation by L-ON in unchelexed Minimal A. Addition of 50 μM ZnSO4 overcomes biofilm inhibition due to 1 μM TPEN. *P < 0.05 vs. untreated by one-way ANOVA with Dunnett’s multiple comparisons test. (TIF) [file ppat.1008707.s011.tif]

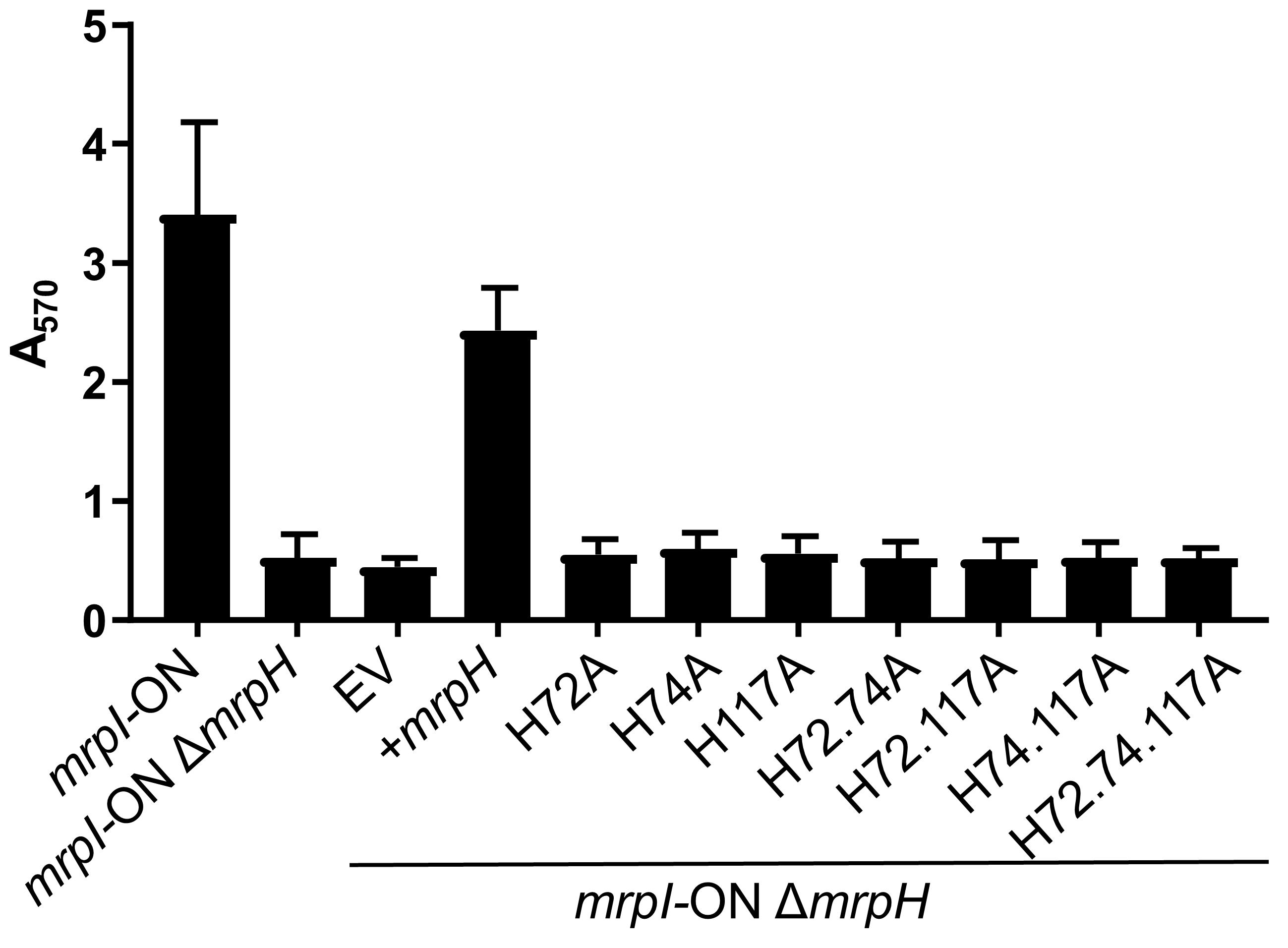

Supplement: S8 Fig — Compare with Fig 11. Biofilm formation by an MR/P locked-ON mutant, a locked-ON mrpH double mutant, and the double mutant complemented with wild-type or mutated mrpH expressed from plasmid pGEN-Pmrp-mrpH (+mrpH). Mutating any of the Zn-coordinating histidine residues (His 72, His 74, His 117) to alanine, alone or in combination, completely abolished P. mirabilis biofilm formation. pGEN-Pmrp-luxCDABE (EV) is a negative control plasmid and has luciferase genes under the control of the native mrp promoter. All columns, including mrpI-ON positive control, are P < 0.0001 vs. pGEN-Pmrp-mrpH by one-way ANOVA with Dunnett’s multiple comparisons test. (TIF) [file ppat.1008707.s012.tif]

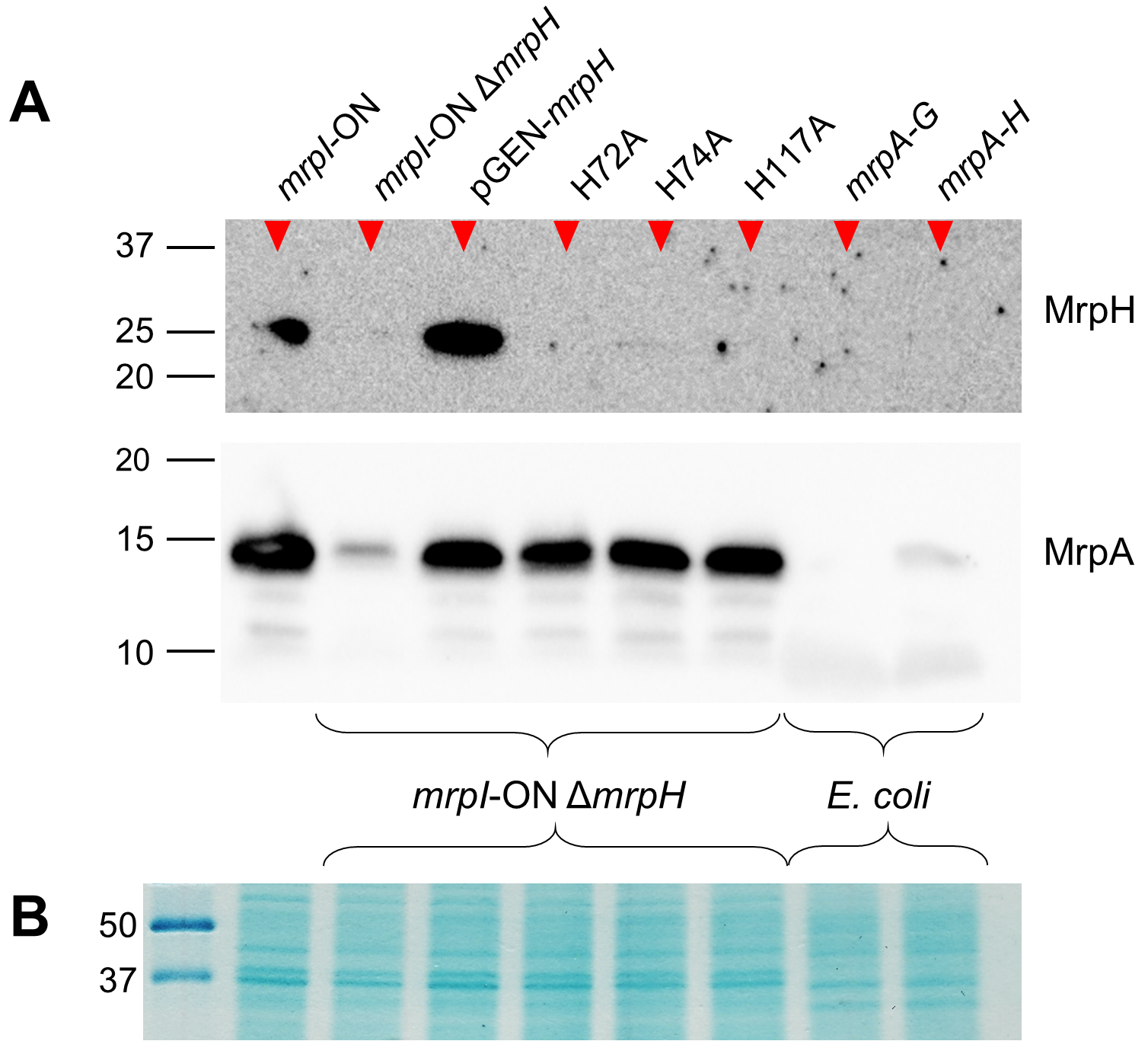

Supplement: S9 Fig — (A) Whole cell lysates of 48h static cultures were subjected to acid treatment to dissociate fimbrial subunits and separated by SDS-PAGE. Following detection of MrpH, blots were stripped and reprobed with anti-MrpA antibodies. Lanes 1–6 are P. mirabilis; lane 1 is mrpI-ON, and lanes 2–6 are mrpI-ON ΔmrpH. Lane 2, no plasmid; lane 3, pGEN-Pmrp-mrpH; lanes 4–6, site-directed mutants as indicated. Lanes 7–8 are E. coli BW25113ΔfimA containing either plasmid pXL4401 (mrpA-G) or pXL1305 (mrpA-H). Molecular size markers in kilodaltons are shown on the left. (B) a replicate polyacrylamide gel was stained with Coomassie blue to assess protein loading. (TIF) [file ppat.1008707.s013.tif]

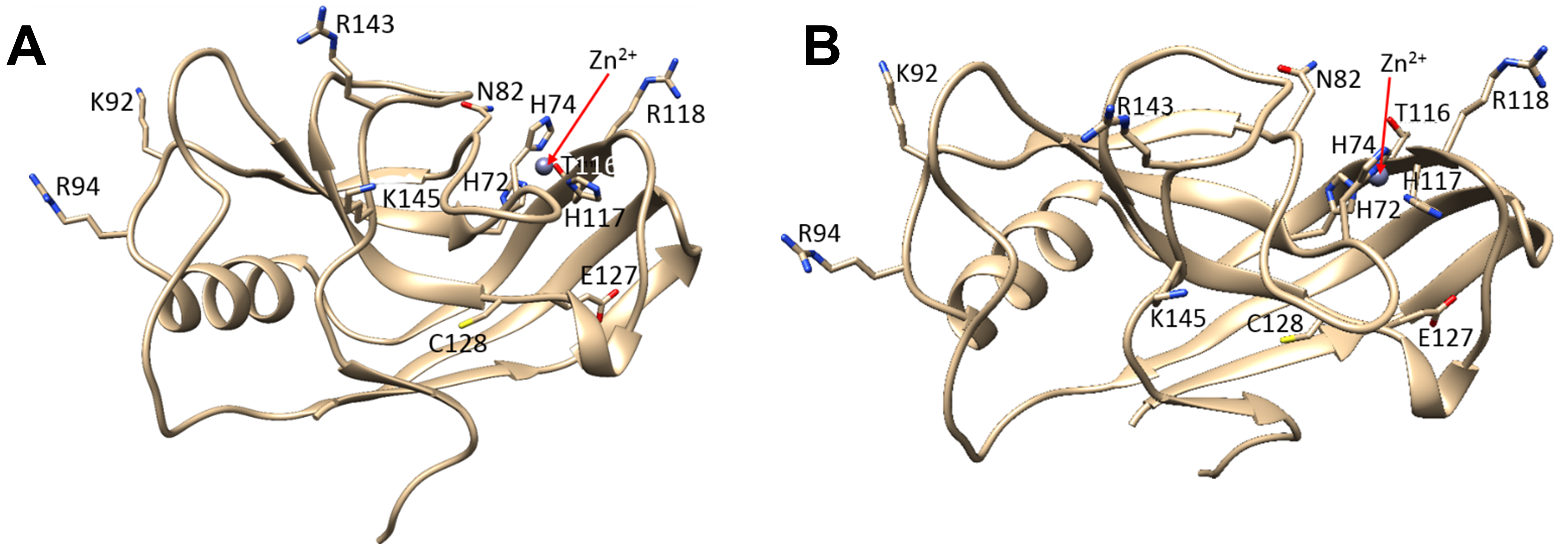

Supplement: S10 Fig — MrpHntd is shown as a ribbon with targeted residues shown as stick models and labeled. (A) side view, (B) top view. (TIF) [file ppat.1008707.s014.tif]

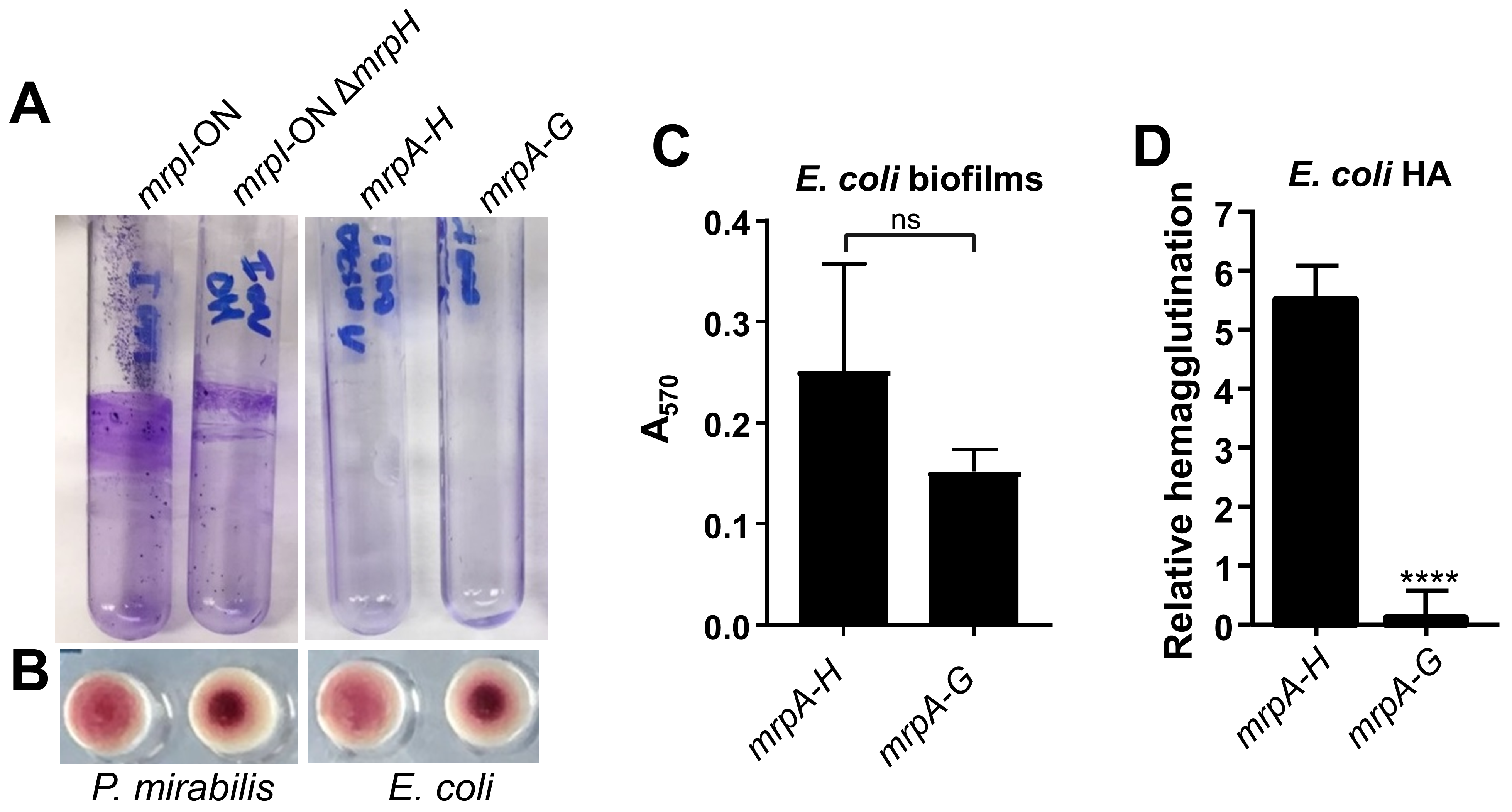

Supplement: S11 Fig — (A) Biofilm formation by P. mirabilis HI4320 mutants (left) and E. coli BW25113ΔfimA with plasmids pXL1305 (mrpA-H) or pXL4401 (mrpA-G) (right). (B) HA by the same P. mirabilis and E. coli strains (representative experiments). (C and D) Quantification of E. coli biofilm formation and HA, respectively (n = 6 independent replicates). Panel D shows the same data as in Fig 12B and is included here to facilitate comparison. (TIF) [file ppat.1008707.s015.tif]
